# Leaf nutrient content and transcriptomic analyses of endive (*Cichorium endivia*) stressed by downpour-induced waterlog reveal a gene network regulating kestose and inulin contents

**DOI:** 10.1038/s41438-021-00513-2

**Published:** 2021-05-01

**Authors:** Giulio Testone, Anatoly Petrovich Sobolev, Giovanni Mele, Chiara Nicolodi, Maria Gonnella, Giuseppe Arnesi, Tiziano Biancari, Donato Giannino

**Affiliations:** 1 Institute for Biological Systems, National Research Council (CNR), Via Salaria Km 29,300 - 00015 Monterotondo, Rome, Italy; 2 Institute of Sciences of Food Production, CNR. Via G. Amendola 122/O - 70126, Bari, Italy; 3 Enza Zaden Italia, Strada Statale Aurelia km. 96.400 – 01016 Tarquinia, Viterbo, Italy

**Keywords:** Transcriptomics, Metabolomics, Regulatory networks, Flooding, RNA sequencing

## Abstract

Endive (*Cichorium endivia* L.), a vegetable consumed as fresh or packaged salads, is mostly cultivated outdoors and known to be sensitive to waterlogging in terms of yield and quality. Phenotypic, metabolic and transcriptomic analyses were used to study variations in curly- (‘Domari’, ‘Myrna’) and smooth-leafed (‘Flester’, ‘Confiance’) cultivars grown in short-term waterlog due to rainfall excess before harvest. After recording loss of head weights in all cultivars (6-35%), which was minimal in ‘Flester’, NMR untargeted profiling revealed variations as influenced by genotype, environment and interactions, and included drop of total carbohydrates (6–50%) and polyols (3–37%), gain of organic acids (2–30%) and phenylpropanoids (98–560%), and cultivar-specific fluctuations of amino acids (−37 to +15%). The analysis of differentially expressed genes showed GO term enrichment consistent with waterlog stress and included the carbohydrate metabolic process. The loss of sucrose, kestose and inulin recurred in all cultivars and the sucrose-inulin route was investigated by covering over 50 genes of sucrose branch and key inulin synthesis (*fructosyltransferases*) and catabolism (*fructan exohydrolases*) genes. The lowered expression of a sucrose gene subset together with that of *SUCROSE:SUCROSE-1-FRUCTOSYLTRANSFERASE* (*1-SST*) may have accounted for sucrose and kestose contents drop in the leaves of waterlogged plants. Two anti-correlated modules harbouring candidate hub-genes, including *1-SST*, were identified by weighted gene correlation network analysis, and proposed to control positively and negatively kestose levels. In silico analysis further pointed at transcription factors of GATA, DOF, WRKY types as putative regulators of *1-SST*.

## Introduction

Curly- and smooth- leafed endives (*Cichorium endivia var. crispum* and *var. latifolium*) are consumed worldwide as fresh or minimally processed salads that are sources of healthy nutrients^1^ and good-profit greens in the export of EU major producers such as Spain, France and Italy (TrendEconomy, http://trendeconomy.com). The Italian cultivation of endives (http://dati.istat.it/) occurs mostly in open field (97% of 8426 ha and yield of 1886738 q in 2019) in autumn-winter cycles because it is low input and cold-tolerant crop^2^. Precipitation excess and soil inadequate drainage cause root waterlogging that dramatically affect product yield and quality. Waterlog stress is characterized by lower oxygen and nutrient availability (e.g. leaching of nitrogen fertilizers) together with enhanced plant susceptibility to diseases favoured by congenial conditions for pathogens^3^.

In outdoor cultivation, oxygen deficiency directly affects the root while the shoot remains oxygenated, underground and aerial organs react differently with anatomical and metabolic modifications^4^. Overall, the waterlog impact on plant metabolism varies with severity, timing and duration of stress and with genotype tolerance^5^. Communication from anoxic root to shoot occurs via xylem^6^, throughout root signals such as tricarboxylic acids (TCA)^7^ and hormones^8^. Leaf responses include stomatal closure and non-stomatal metabolic alterations (e.g. oxidative stress) that lead to decreased CO_2_ incorporation and net photosynthesis drop, while the bleaching is recurrently associated with chlorophyll loss and senescence. Frequently, leaves of sensitive crops undergo stronger alteration in sugar, amino acids and TCA metabolism. On the contrary, tolerant species, which are more efficient in carbohydrate utilization, the show raised levels of fumarate, γ-aminobutirric acid and alanine belonging to routes tailored to compensate anoxia damages^9^.

Inulin has prebiotic and healthy properties, has been used in food and non-food applications, and frequently extracted from the taproot of chicory (*C. intybus* var. *sativum*), which has been a model system to study or modify inulin metabolism^10^. Chemically, root chicory inulin is a fructan-type polymer made of fructose subunits linked by ß-2,1 bonds ending with a α-linked glucose (reduced form, lacking the terminal glucose, can also be found). The degree of polymerization (DP) of the subunits varies from 2 to 60, and its partial enzymatic hydrolysis products (DP 2–8) are also named oligofructose or fructo-oligosaccharides (FOS). Being water soluble, fructans can localize in vacuole, apoplast and xylem, and mainly represent energy storage with extended roles in stress protection^11^. In leaves, inulin occurs at much lower levels and DP than roots; for instance, witloof leaves contain about 14 vs 1300 mg g^−1^ dry weight and 3–5 vs 18–20 DP compared to roots; relatedly, endive leaves were showed comparable levels with witloof^12^. Finally, inulin content of chicory leaf increases with maturity^13^ or by sucrose induction^11^. Given the importance of vegetable intake in human diets, more information on inulin and FOS of *Cichorioideae* salads is envisaged.

In chicory, inulin is under control of the biosynthetic and sequentially acting enzymes (fructan active enzymes, FAZYs) sucrose:sucrose 1-fructosyltransferase (1-SST) and fructan:fructan 1-fructosyltransferase (1-FFT), and of the depolymerizing 1-fructan exohydrolases (1-FEH). The 1-SST transfers fructose from donor sucrose to second sucrose, which accepts the fructose to produce free glucose and 1-kestotriose. The latter is a fructose donor that allows the 1-FFT elongating the inulin chain and releasing free sucrose. The 1-FEH specifically acts on ß-2,1 bonds, breaking fructans into sucrose and fructose, and three isoforms named I, IIa and IIb occur in chicory^14,15^. The genes of the above-mentioned enzymes were characterised at the genomic, phylogenetic and expression levels, and positive correlation among mRNA abundance, enzyme activity and end-product strongly supported tight control at the transcriptional level^16^.

Few studies have focused on metabolic changes of *Cichorioideae* salad heads as challenged by waterlog^17,18^. Recently, integrated omics technologies have been helpful to get insights in nutrient, physiological and molecular aspects of endives^19^. In this work, untargeted metabolite profiling by NMR and transcriptomic analyses were used to quantify the changes of thirty compounds in leaves of endives harvested after natural waterlog, address the transcriptomic response variability among smooth- and curly- cultivars, characterise gene networks that regulate inulin contents, and provide indication on endive-genotypic performance in case of precipitation excess.

## Results and discussion

### Rainfall excess and waterlog affect the productivity of smooth- and curly-leafed endives

**Fig. 1:**
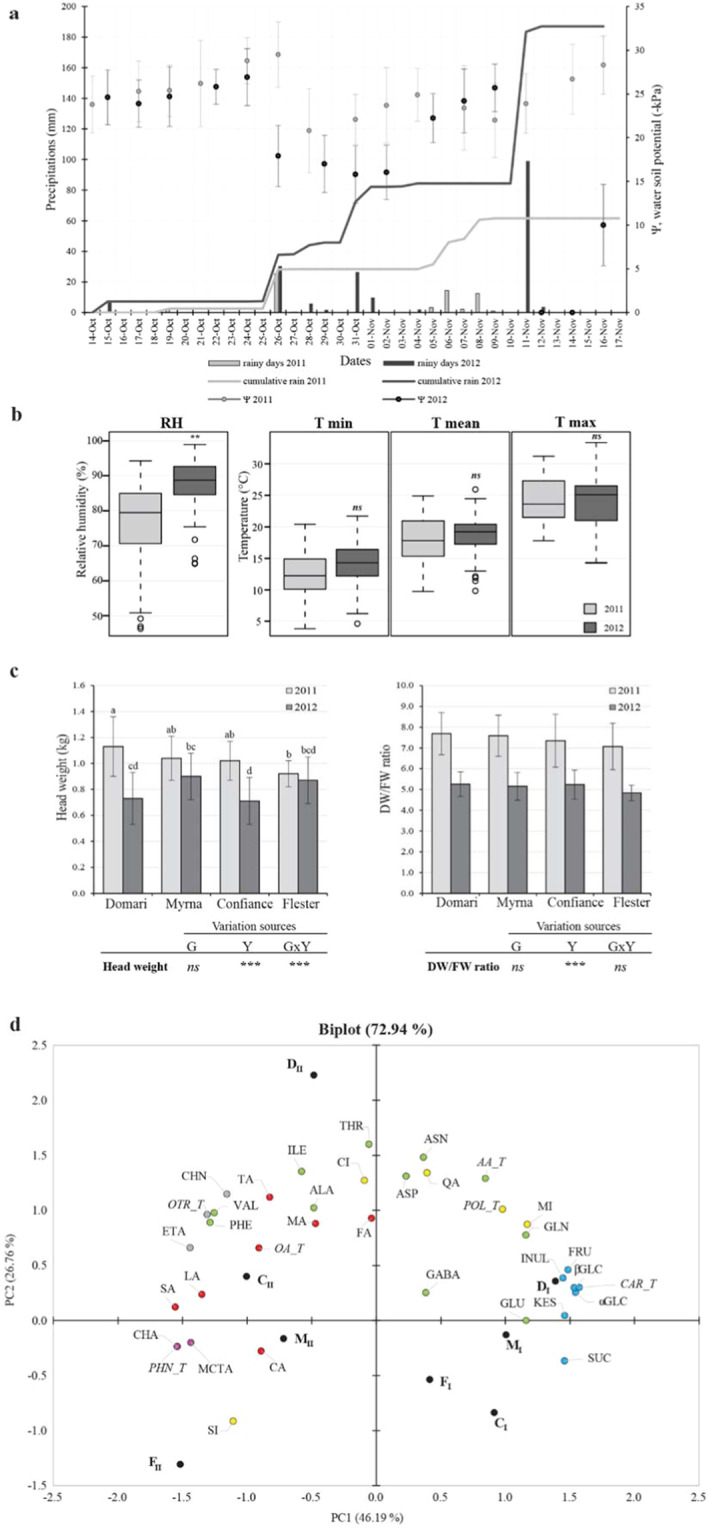
Climate condition, leaf parameters and metabolite explorative analysis. **a**–**b** Climate data during cultivation in 2011 and 2012. **a** Cumulative rainfall and rainy days (left *y*-axis), and soil humidity (right *y*-axis) one month before harvest. **b** Boxplot of relative humidity (RH) and temperatures (T). Significance by Student’s *t*-test: *, ** = significant at *P* ≤ 0.05, 0.01, respectively; ns, non-significant. **c**–**d** Product parameters and principal component analysis of leaf metabolites (PCA). **c** Histograms of head weights (left panel) and dry vs fresh weight ratios of leaves (right panel). Effects of genotype (G), year (Y) and interaction (G×Y) by two-way ANOVA are below each panel. *, **, *** = significant at *P* ≤ 0.05, 0.01 and 0.001 respectively; ns non-significant. **d** PCA biplot shows the spatial distribution of 30 hydrosoluble compounds in curly Domari and Myrna (D, M) and smooth Confiance and Flester (C, F) cultivated in Lazio in 2011 (subscript I) and 2012 (subscript II). Sugars (blue circles): GLC, Glucose; FRU Fructose, SUC Sucrose, KES Kestose, INUL inulin, CAR-T total carbohydrates. Amino acids (green): ALA Alanine, ASN Asparagine, ASP Aspartic acid, GABA γ-Aminobutyric acid, GLN Glutamine, GLU Glutamic acid, ILE Isoleucine, PHE Phenylalanine, THR Threonine, VAL Valine, CA Citric acid, AA-T total amino acids. Organic acids (red): FA Fumaric acid, MA Malic acid, LA Lactic acid, SA Succinic acid, TA Tartaric acid, OA-T total organic acids. Polyols (yellow): CI Chiro-inositol, MI Myo-inositol, QA Quinic acid, SI Scyllo-inositol, POL-T total polyols. Phenols (purple): CHA Chicoric acid, MCTA Monocaffeoyl tartaric acid, PHN-T total phenols. Others (dark grey): CHN Choline, ETA Ethanolamine, OTR-T total other compounds

Two summer-fall productive cycles of endives were carried out at comparable conditions on the same parcel (Table S1) in 2011 (Y1) and 2012 (Y2). Climate analyses of Y2 vs Y1 showed significant differences in rainfall (Fig. 1a) and humidity but not in temperature values (Fig. 1b). During the entire cultivation cycle in Y2 (Table S2), a doubled number of rainy days together with heavy rainfall lead to +233% rain surplus compared to Y1 (up to +245% within 15 days before harvest). Consistently, relative humidity in Y2 was at least 10% higher than Y1 (Fig. 1b). The rainfall excess in Y2 (11-12/11/2012) caused waterlogging (soil oversaturation, 0 kPa) that involved only the root-zone^20^ and was recorded to last 72 h until soil draining (field capacity; 10kPa) was restored. The variation of head weights (HW) and the dry vs fresh weight ratio (DW/FW) of leaves were analysed (Fig. 1c) with respect to the cultivation year (Y) and genotype (G). The Y and GxY effects were significant on HW, while only Y was significant for DW/FW, and no variation was due to G. In Y2, the HW loss was −35%, −14%, −30% and −6% respectively in Domari, Myrna, Confiance and Flester (D, M, C and F); the DW/FW loss was −29% in M and −32% in the others. Weight loss of all cultivars recalled behaviour of crops highly susceptible to waterlog^21^, even though endive harvest occurred in a presumed recovery stage. In indoor experiments, root chicory was shown to compensate intermittent flood stress by increasing leaf number and modifying root shape^22^. Here, it is speculated that the different sensitivity of endives might rely on the root system made of abundant fibrous roots prevailing over a small taproot in endive, while the opposite occurs in industrial chicory. Finally, indoor-induced waterlog caused decrease of lettuce biomass in a cultivar-dependent manner^17^, while GxY effects were most significant for endives in our conditions.

### Untargeted metabolic profiling by NMR

Metabolic phenotyping of D, M, C and F leaves was achieved by NMR assignment of 30 hydrosoluble compounds (Table S3), grouped into carbohydrates, organic acids, amino acids, polyols, phenylpropanoids and other compounds, and quantifications (Table 1) referred to Y1 and Y2. The data set was explored by PCA (Fig. 1d), the PC1 and 2 respectively explained 46.2 and 26.8% of the total variance. Specifically, the PC1 separated the Y1 from Y2 products (positive and negative values of D_I_, M_I_, F_I_ and C_I_ vs D_II_, M_II_, F_II_ and C_II_), and pointed at total carbohydrates (GLC, KES, INUL, SUC and FRU) and phenols (CHA, MCTA) as the most diverging variable groups. Moreover, the biplot diagonal from bottom left to up right (not shown) depicts the GxY effects due to PC1 and 2 contributions. The metabolic (and transcriptomic) analysis was narrowed to edible product; consequently, it could not directly address variations/responses that had occurred in root, stem and organ interplays.

**Table 1:** Content variation of hydrosoluble compounds in four endive cultivars from two growth cycles

	Contents (mg g^−1^ dry weight)								Variation sources		
	Year I				Year II						
Comp.	Domari	Myrna	Confiance	Flester	Domari	Myrna	Confiance	Flester	G	Y	G×Y
αGLC	40.36 ± 9.83a	40.77 ± 5.28a	40.73 ± 6.29a	37.95 ± 6.88a	31.93 ± 4.2ab	18.97 ± 4.17 cd	25.91 ± 7.67bc	16.16 ± 4.35d	ns	***	*
βGLC	77.06 ± 17.56a	76.81 ± 9.67a	76.44 ± 11.52a	72.13 ± 13.67a	61.92 ± 9.31ab	35.92 ± 8.29 cd	49.75 ± 14.87bc	31.14 ± 7.41d	ns	***	**
FRU	148.21 ± 35.64	144.63 ± 18.82	145.61 ± 19.51	142.64 ± 26.08	134.28 ± 16.81	93.98 ± 21.62	112.22 ± 31.92	84.47 ± 17.55	ns	**	ns
INUL	2.59 ± 0.42a	1.99 ± 0.44ab	1.59 ± 0.37bc	1.30 ± 0.30 cd	1.58 ± 1.00bc	0.69 ± 0.30de	0.57 ± 0.37e	0.80 ± 0.41de	***	***	*
KES	8.60 ± 2.13a	8.46 ± 1.97a	4.39 ± 1.10b	4.22 ± 0.77b	3.24 ± 1.36bc	1.31 ± 0.75 cd	0.85 ± 0.42d	2.08 ± 0.87 cd	***	***	***
SUC	34.65 ± 5.97a	25.22 ± 4.27b	28.73 ± 6.61ab	22.53 ± 2.22bc	16.23 ± 3.45 cd	16.09 ± 4.22 cd	9.59 ± 3.12d	17.9 ± 3.81c	***	***	***
***CAR-T***	*311.47* ± *64.11*	*297.89* ± *37.16*	*297.49* ± *40.68*	*280.77* ± *47.75*	*249.19* ± *31.02*	*166.97* ± *34.13*	*198.88* ± *57.74*	*145.53* ± *35.98*	*ns*	***	*ns*
CA	3.91 ± 1.41	3.34 ± 0.82	6.25 ± 1.47	4.46 ± 1.15	5.18 ± 2.78	3.72 ± 1.28	6.58 ± 1.67	6.52 ± 1.83	***	ns	ns
FA	0.09 ± 0.03	0.12 ± 0.03	0.08 ± 0.01	0.11 ± 0.02	0.13 ± 0.02	0.13 ± 0.12	0.09 ± 0.02	0.08 ± 0.01	ns	ns	ns
LA	0.05 ± 0.03	0.07 ± 0.03	0.04 ± 0.03	0.06 ± 0.03	0.08 ± 0.04	0.08 ± 0.05	0.07 ± 0.03	0.09 ± 0.05	ns	ns	ns
MA	19.69 ± 1.77b	19.15 ± 1.74b	20.26 ± 2.16b	18.92 ± 1.83b	24.02 ± 1.69a	18.6 ± 2.95b	19.11 ± 2.23b	21.52 ± 2.64ab	ns	ns	**
SA	0.19 ± 0.06d	0.20 ± 0.03d	0.26 ± 0.06d	0.29 ± 0.06d	0.55 ± 0.08b	0.41 ± 0.06c	0.48 ± 0.11bc	0.70 ± 0.09a	**	***	***
TA	1.29 ± 0.27b	0.92 ± 0.17b	1.45 ± 0.31b	0.94 ± 0.21b	2.85 ± 1.05a	1.32 ± 0.37b	3.01 ± 0.45a	1.40 ± 0.35b	**	***	***
***OA-T***	*25.21* ± *2.45cde*	*23.79* ± *1.88e*	*28.34* ± *2.76bcd*	*24.79* ± *2.6de*	*32.8* ± *2.93a*	*24.24* ± *3.89de*	*29.34* ± *3.14abc*	*30.31* ± *3.35ab*	**	*ns*	**
ALA	0.60 ± 0.11bc	0.73 ± 0.12ab	0.53 ± 0.12c	0.55 ± 0.12bc	0.73 ± 0.05ab	0.71 ± 0.13abc	0.81 ± 0.22a	0.55 ± 0.08bc	**	***	**
ASN	6.01 ± 1.30bc	6.17 ± 1.09bc	5.21 ± 1.31bc	4.09 ± 1.12 cd	8.61 ± 3.18a	4.92 ± 2.02bc	7.11 ± 2.12ab	1.87 ± 0.36d	*	ns	***
ASP	1.54 ± 0.34a	1.20 ± 0.17ab	1.09 ± 0.21b	1.39 ± 0.19ab	1.55 ± 0.17a	1.26 ± 0.26ab	1.36 ± 0.13ab	1.13 ± 0.28b	***	ns	**
GABA	0.86 ± 0.24bc	1.49 ± 0.29a	0.57 ± 0.18c	0.98 ± 0.17bc	0.94 ± 0.20bc	1.07 ± 0.56ab	0.75 ± 0.12bc	0.77 ± 0.18bc	***	ns	*
GLN	6.13 ± 2.22ab	7.66 ± 2.34a	3.05 ± 1.15 cd	2.30 ± 0.80d	4.88 ± 2.35bc	2.52 ± 1.56 cd	2.72 ± 1.07 cd	0.69 ± 0.10d	***	ns	***
GLU	2.69 ± 0.85a	1.71 ± 0.59bc	2.34 ± 0.49ab	1.76 ± 0.47bc	1.81 ± 0.32bc	1.96 ± 0.45abc	1.44 ± 0.14c	1.61 ± 0.28bc	***	***	**
ILE	0.15 ± 0.04b	0.13 ± 0.02b	0.08 ± 0.03c	0.16 ± 0.03b	0.27 ± 0.03a	0.15 ± 0.02b	0.15 ± 0.01b	0.15 ± 0.02b	***	***	***
PHE	0.13 ± 0.04c	0.13 ± 0.03c	0.12 ± 0.04c	0.24 ± 0.04b	0.39 ± 0.04a	0.27 ± 0.05b	0.28 ± 0.04b	0.27 ± 0.03b	***	***	***
THR	0.45 ± 0.13bcd	0.47 ± 0.06bc	0.32 ± 0.06de	0.31 ± 0.05de	0.73 ± 0.19a	0.41 ± 0.12 cd	0.56 ± 0.13b	0.23 ± 0.05e	***	***	***
VAL	0.27 ± 0.07ef	0.28 ± 0.04de	0.20 ± 0.06 f	0.3 ± 0.03cde	0.45 ± 0.05a	0.38 ± 0.07ab	0.37 ± 0.03bc	0.36 ± 0.05bcd	***	***	**
***AA-T***	*18.85* ± *4.13ab*	*19.96* ± *3.82ab*	*13.51* ± *2.37c*	*12.08* ± *1.78* *cd*	*20.36* ± *5.14a*	*13.67* ± *3.51c*	*15.53* ± *3.37bc*	*7.63* ± *0.96d*	***	*ns*	***
CHA	0.87 ± 0.41de	0.78 ± 0.37de	0.42 ± 0.28e	1.16 ± 0.68cde	1.72 ± 0.49 cd	2.10 ± 0.98bc	3.05 ± 1.31ab	4.03 ± 1.08a	ns	***	***
MCTA	0.08 ± 0.02b	0.14 ± 0.08b	0.07 ± 0.04b	0.07 ± 0.03b	0.16 ± 0.06ab	0.19 ± 0.17ab	0.18 ± 0.08ab	0.29 ± 0.14a	ns	*	*
***PHN-T***	*0.95* ± *0.42de*	*0.93* ± *0.37de*	*0.49* ± *0.29e*	*1.23* ± *0.69cde*	*1.88* ± *0.54* *cd*	*2.29* ± *0.91bc*	*3.23* ± *1.36ab*	*4.32* ± *1.21a*	*ns*	***	***
CI	0.66 ± 0.27a	0.28 ± 0.06 cd	0.39 ± 0.15bcd	0.21 ± 0.07d	0.77 ± 0.02a	0.59 ± 0.08ab	0.44 ± 0.12bc	0.32 ± 0.07 cd	***	ns	*
MI	5.76 ± 0.58a	4.19 ± 0.6 cd	5.05 ± 0.34abc	5.00 ± 0.39abc	5.34 ± 0.85ab	3.09 ± 1.53de	4.34 ± 0.58bc	2.46 ± 0.26e	***	ns	***
SI	0.19 ± 0.05	0.17 ± 0.05	0.38 ± 0.07	0.45 ± 0.09	0.25 ± 0.05	0.28 ± 0.31	0.47 ± 0.09	0.69 ± 0.10	***	ns	ns
QA	0.38 ± 0.06	0.26 ± 0.06	0.28 ± 0.04	0.24 ± 0.05	0.44 ± 0.04	0.27 ± 0.09	0.24 ± 0.03	0.24 ± 0.03	***	ns	ns
***POL-T***	*7.00* ± *0.88a*	*4.89* ± *0.69cde*	*6.10* ± *0.46abc*	*5.89* ± *0.50abc*	*6.80* ± *0.83ab*	*4.23* ± *1.97de*	*5.49* ± *0.60bcd*	*3.71* ± *0.28e*	***	*ns*	*
CHN	1.20 ± 0.11 cd	1.21 ± 0.10 cd	1.13 ± 0.09d	1.30 ± 0.09 cd	1.83 ± 0.13a	1.51 ± 0.15b	1.73 ± 0.14a	1.38 ± 0.14bc	*	***	***
ETA	0.80 ± 0.22 cd	0.73 ± 0.12d	0.84 ± 0.16 cd	0.96 ± 0.16bc	1.22 ± 0.07a	1.13 ± 0.05ab	1.20 ± 0.08a	1.08 ± 0.06ab	**	***	**
***OTR-T***	*2.01* ± *0.27ef*	*1.94* ± *0.17* *f*	*1.97* ± *0.18ef*	*2.26* ± *0.20de*	*3.05* ± *0.19a*	*2.63* ± *0.19bc*	*2.93* ± *0.20ab*	*2.47* ± *0.18* *cd*	**	***	***

Italic entries indicate significant GxY interactions and letters except for the CAR-T (total carbohydrates) because it is not significant

Study of effects by genotype and year as sources of variation using data from cultivars produced by outdoor winter cultivation cycles (2011, 2012) in Lazio. G, genotype; Y, year; G × Y, genotype: year interaction. Significance letters refer to G × Y effect; ns, non-significant; *, **, *** = significant at *P* ≤ 0.05, 0.01 and 0.001 respectively. Mean conversion factors into mg g^−1^ FW for Domari, Myrna, Confiance, and Flester are respectively: 13.00, 13.17, 13.60, 14.13 in 2011, and 19.04, 19.42, 19.13 and 20.69 in 2012. As for compound abbreviations, see legend of Fig. 1d

#### Carbohydrates

The ANOVA deepened data exploration, and min to max value ranges hereafter refer to mg g^−1^ dry weight. Synoptically (Table 1), FRU (84.5–148.2) was the most abundant carbohydrate, followed by α- and β-GLC (16.2–40.8 and 31.1–77.1, respectively), SUC (9.6–34.6), and by KES and INUL (0.8–8.6 and 0.6–2.6, respectively). Y influenced the content variation of all sugars; the G effect was specific on INUL, KES and SUC, while the GxY acted on all of them except for FRU. The CAR-T loss in Y2 was −20% in D, −33% in C, −44% in M, and −48% in F as compared to Y1 production. Focusing on KES and INUL contents, in Y2 they dropped intensely and differentially according to cultivars (INUL, −38% in D and F to −65% in M and C; KES, over −50% in D and F and over −80% in M and C), and their contents showed a strong positive correlation (Fig. S1). From now on, discussion themes will mainly refer to literature on endive using conversion factors (Table 1 legend reports DW to FW formula) and to leaf metabolism as consequence of waterlog. Two sources^23,24^ reported on GLC/FRU/SUC ranges in endives that were consistent with this work. Moreover, inulin ranges were measured in outdoor curly endive^12^ and consistent with KES + INUL levels of D and M curly types. Regarding flavour (Table S4), the computed sucrose sweetness equivalency (SSE) was mainly affected by GLC, FRU and SUC variations, given that INUL and KES are only 2–6% of total sugars and have low relative sweetness power (0.10–0.22 and 0.33 vs SUC as unit reference). Y and G×Y (but not G) influenced SSE just as CAR-T contents, and SSE decreased up to −46% in M and F in the rainier Y2. Moreover, no significant association occurred between sugar content/SSE and leaf phenotype. In addition, considering the INUL + KES contents of 0.07–0.86 mg g^−1^ FW, one hundred gram serve of endive provides much <20 g inulin that is the human well-tolerated daily dose^25^. G effects are known for dietary fibre of endives^26^ consistently with this work. Waterlog susceptible species usually show decreased levels of leaf GLC/FRU/SUC oppositely to tolerant ones^27,28^. The carbohydrate drop in endives confirms vulnerability to soil flooding as observed in taproot chicory^22^. However, SUC levels undergo complex variations since accumulation and loss were respectively measured in soy^29^ and chicory leaves^22^ in response to waterlog. Finally, sugar export from leaves is necessary to sustain the increased glycolysis for ATP production in hypoxic roots^30^, supported by the evidence of concurrent decrease of SUC in leaf and increase in the phloem sap of hypoxic plants^31^. Contextually, we propose that the carbohydrate loss in endives may derive from both reduced synthesis and enhanced export from leaves responding to short-term floods.

#### Organic acids

MA, CA and TA showed the highest levels (Table 1), and G, Y and GxY effects were significant on SA and TA, but not on FA and LA; CA was affected only by G and MA only by GxY. The total mean levels raised 2–4% in M and C, 22–30% in F and D in Y2. The MA and CA ranges reported for a curly type were comparable to this work, while SA was measured as 10-fold higher^23^ (here 0.001–0.003 g 100 g^−1^ FW). We were unable to retrieve literature data on FA, LA and TA ranges in endives, however, our values (6–150 mg 100 g^−1^ FW) fell in the ample variations of leaves (0.55–685.28 and 750–3000 mg 100 g^−1^ FW) of numerous species^1,32^. Shoots of flooded crops can show an increase in fermentation metabolites such a SA^6^ and LA^4^, typically accumulated in hypoxic roots, suggesting the TCA and lactate routes may be activated in leaves. However, LA and SA enrichment in sap imported from waterlogged roots^6^ cannot exclude channelling into leaves. OA metabolism of leaves responding to flooded roots varies with species and stress duration, e.g., chicory leaves showed MA and CA accumulation^22^ contrary to soy and Arabidopsis responses^4,6^. Regarding endives, the SA increase (85–189%) in all cultivars from the rainier Y2 is consistent with waterlog effects; however, key genes of anaerobic pathways were not triggered in leaves (see the following sections) and other routes must have been involved. Finally, post-waterlogging endives shared TA raised levels (49–120%), which has not been reported to our knowledge so far.

#### Amino acids

ASN and GLN had the highest contents followed by GLU and ASP, while the other amino acids (AA) were below one mg g^−1^ DW (Table 1). G and GxY affected the levels of all AA; Y had also strong effects on most AA, though non-significant on ASN, ASP, GLN, GABA. The AA-T level raised by 8–15% in D and C, and decreased by 32-37% in M and F in Y2. Databases on endive (https://fdc.nal.usda.gov; http://www.fao.org) just report mean values that are ca. 10 fold higher than in this work, whereas consistency was found with surveys addressing season and genotype effects in endive^33^. In roots, the AA metabolism develops in complex manner depending on species and stress conditions and is usually characterised by the decrease of most AA^6^ and raise of anoxic responsive ones such as ALA or GABA^4^. The yearly variation of these latter was genotype specific in endive leaves, indeed, D and C showed gain in GABA and ALA (+9 and 32%; +22 and 53%, respectively) differently from M and F (−21 and −28%; −3 and 0% respectively). Finally, most endives showed drop (up to −42%) of NO_3_^-^ content in Y2 vs Y1 harvest (Table S5). This is may be due to N depletion in waterlogged soils (e.g. by microbe denitrification, leaching), root damaging, and impairment of nitrate efficiency use as suggested by the altered gene expression in the “nitrate assimilation” gene-ontology term (GO:0042128 in Table S6).

#### Phenylpropanoids

They included two hydroxycinnamic acids (HA), CHA showed wide value ranges (0.4–4.0) while MCTA was much less abundant (<0.5), and variations were under significant control of Y and GxY (Table 1). The CHA and MCTA ranges of endives from literature^34^ were consistent with this work (curly: 59.2–159.4 and 6.15–14.4; smooth: 30.8–285.21 and 4.9–20.5 mg kg^−1^ FW). In the rainier Y2, CHA levels leaped from 98 (D) to over 500% (F), the raised levels of its precursor PHE (13–200%) in Y2 further support the triggering of pathways that favour oxidative damage protection (e.g. shikimate and phenylpropanoids).

#### Polyols

MI content (2.4–5.7) exceed CI and SI ones (Table 1), and the total levels dropped in the rainier Y2 (up to −37% in F); all variables were under G but not Y effects, while GxY affected only CI and MI. In literature^24^, endive inositol amounts varied comparably to this work, except for the MI which was over three-fold higher in this work (11.8–44.3 mg 100 g^−1^ FW) and an added value considering its healthy properties^35^. In Y2, the MI decrease was concurrent with its precursor GLU and with CI and SI increase in all cultivars, suggesting that MI conversion into CI and SI might be a specific stress response^36^. We could not retrieve literature data on QA contents in endive, while chicory leaf ranges^37^ were slightly higher than our samples (3.8 ± 0.2 vs 1.1–2.9 mg 100 g^−1^ FW).

#### Others

CHN and ETA contents were affected by G, Y and G×Y (Table 1). Their total levels significantly increased from 9 to 52% in all cultivars in Y2. ETA is the CHN precursor; both are essential dietary nutrients^38^ and literature ranges (https://www.ars.usda.gov) available for CHN in lettuce (6.7–9.9 mg 100 g^−1^ FW) were in agreement with endive. The content increase in Y2 may relate to their functions in preserving membrane integrity and in ROS scavenging during stresses affecting cell osmosis^39^.

### Transcriptomics and the network of carbohydrate pathway

#### Features of endive enhanced transcriptome and annotation

The lettuce genome sequence^40^ was helpful to guide the assembly and enhance a previous transcriptome of endive^19^ to generate a new version (v.2) through a merging strategy (the detailed pipeline is in the materials and methods); Table 2 reports evaluation metrics for each assembly. The v.2 consisted of 49058 sequences characterized by mean contig length, N50 and N90 values of 1439, 1795 and 804 bp, respectively. Moreover, v.2 had a higher proportion of sequences longer than 1000 bp, increased completeness (92.4% vs 89.8%) and single-copy genes (80.6% vs 65.6%), decreased ratio of duplicated (10.8% vs 24.2%), fragmented (2.5% vs 3.9%) and missing (4.9% vs 6.3%) sequences than v.1. In addition, v.2 had the highest value of full- and nearly full-length sequences (37.7% and 72.9%) followed by lettuce genome-guided assembly (LGA) and v.1 (18.7 and 42.9%; 16.7 and 41.7%, respectively). The v.2 metrics outperformed LGA and v.1, except for the read mapping-back ratios that were only slightly lower than v.1. Referring to the lettuce genome, ca. 70% of v.2 transcripts aligned for over 70% in length (Table S7), reflecting the species relatedness and over 98% of mapped transcripts were on the nine lettuce chromosomes (<1.5% on unplaced scaffolds). Eventually, v.2 functional annotation included gene ontology terms for 35658 transcripts (Table S7). The approach of knowledge transfer from model (lettuce) to non-model (endive) species to reconstruct transcriptome of the latter was successful to by-pass, together with the use of de novo assemblers, bottlenecks (e.g. fragmentation, redundancy and chimerism) that affect ortholog/paralog resolutions, gene-expression quantifications and distance matrix construction^41^. Ultimately, the merging strategy was the best to minimize contigs redundancy and produce a higher quality assembly.

**Table 2:** Evaluation metrics of transcriptomes

Parameters	Assembly v1.0^a^	Genome guided	Assembly v2.0
Sequence numbers	84,882	80,473	49,058
General metrics (bp)			
Mean contig length	1214.4	777.5	1438.9
N50	1591	1353	1795
N90	605	289	804
Sequence length ranges (%)			
≤500 bp	19.0	55.8	11.5
501–1000 bp	32.1	18.0	26.3
1001–1500 bp	20.7	11.1	23.9
1501–2000 bp	13.2	7.3	17.4
2001–2500 bp	7.2	3.7	9.8
2501–3000 bp	3.6	1.9	4.9
>3000 bp	4.2	2.2	6.2
Transcriptome size (Mb)	103.1	62.6	70.6
Read mapping back (%)			
Mapped	95.9	90.7	93.4
Proper pair^b^	81.2	77.2	82.9
BUSCO evaluation (%)^c^			
Completeness	89.8	82.0	92.4
Single copy	65.6	62.8	80.6
Duplicated	24.2	19.2	10.8
Fragmented	3.9	4.7	2.5
Missing	6.3	13.3	4.9
Transcript completeness (%)^d^			
Full length	16.7	18.7	37.7
Nearly full length	41.7	42.9	72.9

^a^Previously published *C. endivia* transcriptome assembly^19^

^b^Read pairs mapping to the same transcript

^c^Total BUSCO groups searched were 1440 from the Embryophyta_odb9 database

^d^Percentage of (nearly- and full-length) transcripts with 70–100% alignment coverage versus respective hits in the NCBI Refseq protein dataset

#### Analysis of differentially expressed genes (DEGs)

**Fig. 2:**
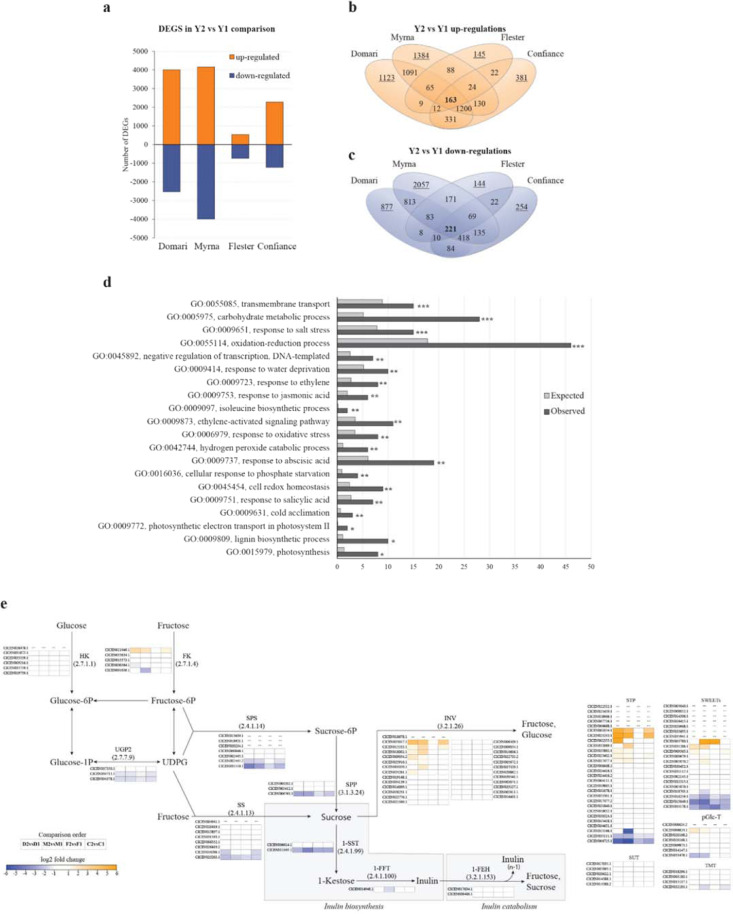
Differentially expressed genes (DEGs) and inulin pathway in waterlogged cultivars. **a** Histogram reporting the total number of DEGs in Domari, Myrna, Flester and Confiance. **b**–**c** Venn diagrams of private and shared DEGs among the four cultivars. The numbers of up- (**b**) and downregulated (**c**) genes are reported. Cultivar-specific DEGS are underlined; genes with common expression trends fall in overlapping areas, core-DEGs were in bold (163 up- and 221 downregulated genes that conserved differential expression pattern independently of genotype). **d** Top 20 GO terms for the biological process category. False discovery rates were calculated. *, **, *** = significant at *P* ≤ 0.05, 0.01 and 0.001 respectively. **e** Genes of sucrose and fructan metabolism. Differential expression patterns in Domari, Myrna, Flester and Confiance in Y2 vs Y1 (D2vsD1, M2vsM1, F2vsF1 and C2vsC1) were highlighted by heatmaps according to log_2_ fold change. Dashes indicate transcripts not expressed in leaves. Glucose-1P glucose 1-phosphate; Glucose-6P, glucose 6-phosphate, UDPG uridine diphosphate glucose, 1-FEH fructan exohydrolase, 1-FFT fructan:fructan-1 fructosyltransferase, 1-SST sucrose:sucrose-1 fructosyltransferase, FK fructokinase, HK hexokinase, INV invertase, pGlc-T plastidic Glucose translocators, SPP sucrose-phosphate phosphatase, SPS sucrose-phosphate synthase, SS sucrose synthase, STP sugar transporter protein, SUT sucrose transporter, SWEET Sugars Will Eventually be Exported Transporter, TMT tonoplast monosaccharide transporters

Cultivar-dependent transcriptomic variation is reported by comparison of Y2 vs Y1 (Fig. 2). Overall, the number of genes that varied expression was higher in curly than smooth types (6535–8151 genes in Myrna and Domari; 1274–3500 in Flester and Confiance). Specifically, 4005, 4157, 2279 and 541 genes were upregulated and 2530, 3994, 1221 and 733 were downregulated respectively in D, M, C and F (Fig. 2a). The four cultivars differed for the number of private DEGs (underlined values in Fig. 2b and c) supporting the activation of cultivar-specific pathways in stress response; Flester showed the DEGs lowest number, which might reflect either less susceptibility/rapid recovery to waterlog, consistently with the lesser weight loss than the other cultivars. Totally, 384 transcripts showed a conserved differential expression pattern independently of genotype (163 up- and 221 downregulated, Fig. 2b and c), and they were named core-DEGs. Gene ontology (GO) enrichment analysis revealed that the core-DEGs were over represented in 182 terms (Table S6) from which the top 20 biological processes were pictured in Fig. 2d. As lettuce and endive share common pathogens, the transcriptomic analyses of endive orthologs to lettuce responding to infections (Table S7) were addressed and the comparison between our data with those of transcriptome variations in lettuce-pathogen interactions did not bring out the response of genes^3,42^ or GO terms typically associated to disease^43^. Hence, the data reinforced that stress sources other than waterlog were minimal and waterlog was likely to be the prevailing cause of the “omic” differences, and consistently, many enriched GO terms of endive recur in leaf transcriptional responses of waterlogged plants^8,44^. Indeed, DEG-enriched terms about ethylene, jasmonate, and ABA typically characterize waterlog-induced hormonal responses^8^. For example, the seven responsive DEGS here found included the arabidopsis orthologues ETHYLENE INSENSITIVE 4 (CICEN034832.1; EIN4) and EIN3-BINDING F BOX PROTEIN 1 (CICEN002529.1; EBF1/2) that are reported as necessary for recovery from flooding^45^, and the endive EBF1 upregulation in Y2 was a consistent event with other waterlog stressed crops^46^. Flooded crops exhibit photosynthetic capacity decrease and downregulation of related genes^47^; consistently, the down tuning of genes of the photosystem I (CICEN011929.1/PSAL, CICEN036571.1/PSAB, CICEN036572.1/PSAA) and II (CICEN036543.1/D1 and CICEN036565.1/D2) in flooded endives suggests the occurrence of photosynthesis impairment (which was not specifically addressed). Moreover, considering that photosynthesis inhibition is accompanied by the accumulation of reactive oxygen species, the top enriched “oxidation-reduction process” term (GO:0055114, Fig. 2d) may further reflect the association of the two events in stressed endives. Finally, the transcriptional reprogramming of genes that regulate sugar metabolism^44^ in waterlog-injured plants is here sustained by the scoring of carbohydrate and sucrose metabolic processes terms (GO:0005975 and GO:0005985), which are mostly characterized by sugar gene downregulation together with carbohydrate loss in stressed endives (Fig. 2d and Table S6).

#### Genes and proteins of sucrose metabolism and the route to inulin

The drop of SUC/KES/INUL contents was common to all cultivars in Y2 (Table 1) and the transcriptional variations subtending this event were addressed by assembling the sucrose branch to fructans inclusive of sugar transporters (Fig. 2e). The route to sucrose was accomplished with transcripts of seven major enzyme classes: six hexokinases (HK), five fructokinases (FK), three UTP-glucose-1-phosphate uridylyltransferase (UGP), seven sucrose phosphate synthase (SPS), eight sucrose synthase (SS), three sucrose phosphate phosphatase (SPP), and twenty-six invertases (INV). Though some were not expressed in leaves, the gene members of *UGP* (CICEN034378.1 and CICEN034713.1), *SPS* (CICEN031550.1), *SPP* (CICEN006763.1), *SS* (CICEN025203.1), sucrose transporters (*STP*; CICEN006725.1 and CICEN035111.1) and *SWEET* (CICEN015049.1, CICEN016259.1 and CICEN033178.1) were commonly downregulated in all cultivars of the rainier Y2 (Fig. 2e). In literature, lower SS and SPS activities marked leaves of brassica sensitive varieties after waterlog stress^48^ and, consistently with our data, leaves of waterlogged crops showed reduced expression of sucrose metabolism genes together with sugar content loss^47^. The sucrose to inulin branch included the transcripts of the three major enzymes 1-FFT (CICEN014948.1), 1-SST (CICEN006614.1 and CICEN011043.1) and 1-FEH (1-FEH I, CICEN009400.1; 1-FEH II, CICEN017634.1). Transcripts of all these featured in leaves, qPCR assays validated their expressions (*R*^2^ = 0.796, *p* < 0.001, Fig. S2), and the downregulation of one *1-SST* (CICEN011043.1) common to all cultivars in Y2 may account for KES drop, consistently with the assessed role of 1-SST in *Asteraceae*^49^. Referring to the lettuce genome (Table S7), the endive *1-SSTs* transcripts mapped on chromosome (chr) 9 and 2, the *1-FFT* was on chr2, the *1-FEHs* were close but spatially separated on chr5. These data suggest they were all distinct alleles rather than spliced forms of the same gene.

The inulin enzymes belong to the glycoside hydrolase family 32 (GHF32), and the phylogenetic tree made of *Cichorioideae* GHF32 members branched out into distinct clades (Fig. S3), one encompassed FEH, cell wall and other INV subclades, and the other included 1-FFT/1-SST and vacuolar INV subclades. Specifically, endive proteins fell in the same phyletic groups as chicory and diverged from lettuce. The 1-SST (CICEN011043.1, CICEN006614.1) and 1-FFT (CICEN014948.1) were closest to the respective chicory orthologs, while FEH proteins (CICEN009400.1, CICEN017634.1) fell respectively in the branch of chicory 1-FEH I and 1-FEH IIb. Intriguingly, endive (as well as lettuce) homologs to the chicory 1-FEHIIa could not be found^15^, while, expectedly, endive proteins conserved key domains of chicory GHF32^50^ (WMNDPNG, WSGSAT, RDP and EC in Fig. S4).

#### Gene modules acting on the inulin route

**Fig. 3:**
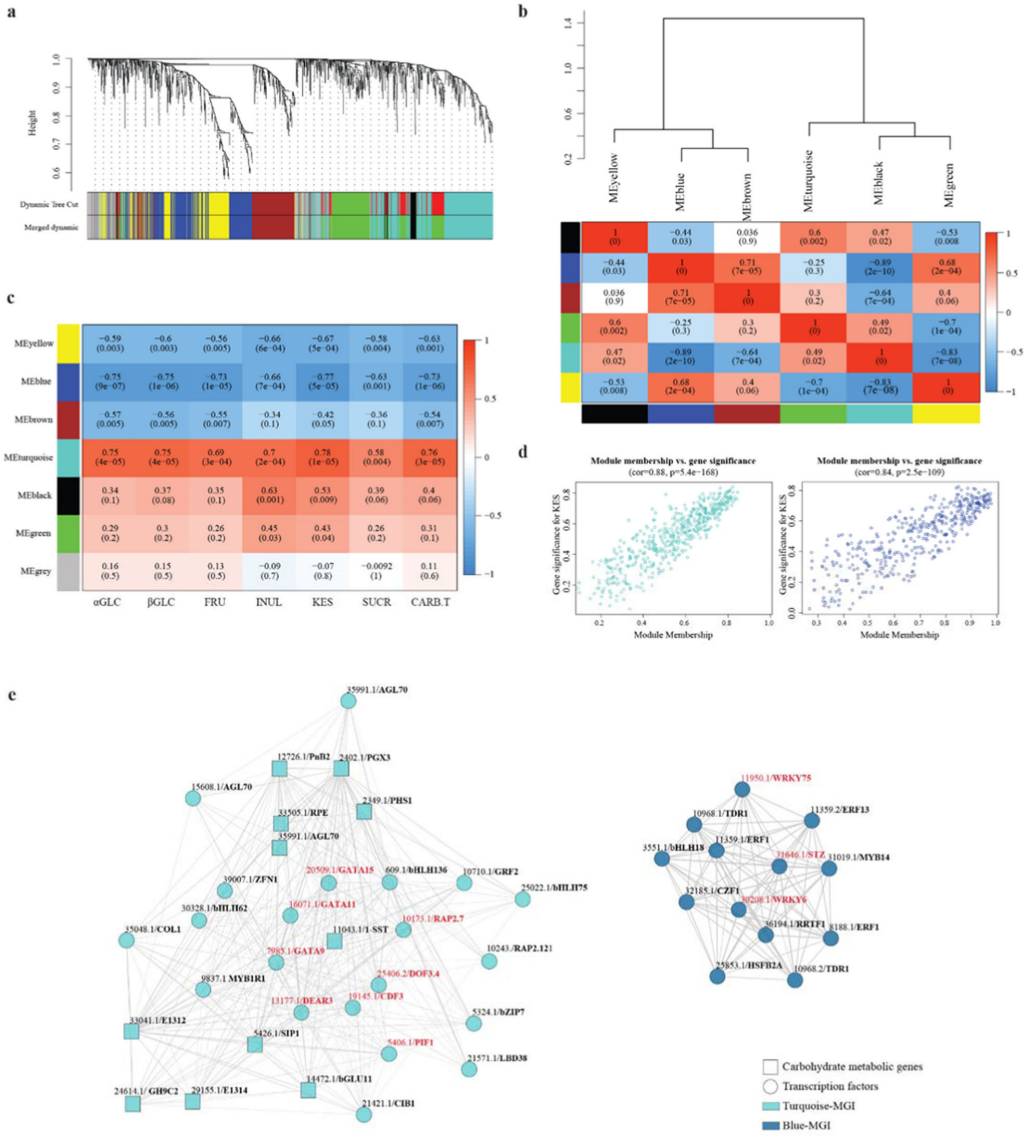
WGCNA and identification of regulatory genes of kestose contents. WGCNA of carbohydrate metabolic process and transcription factor genes from two productions. **a** Cluster dendrogram showing different modules (by colour) of co-expressed genes as identified by the Dinamic Tree Cut algorithm and by merging modules sharing a correlation above 0.75. The grey module included genes that did not belong to any other modules. **b** Hierarchical clustering dendrogram (upper panel) and correlation heatmap (lower panel) of module eigengenes (ME) to examine higher-order relationships between the modules. Correlations and corresponding *p*-values in parenthesis are in coloured boxes. **c** Heatmap of module-carbohydrates correlations. Acronyms of sugars are in Fig. 1d legend. **d** Scatterplots of module membership vs genes significance in the blue and turquoise modules. **e** Cytoscape representation of the module of genes of interest (MGI) within blue and turquoise modules. Edges with weight above a threshold of 0.2 are shown. The transcript numbers and annotations (listed in Table S8) are above the circles (transcription factors) and squares (carbohydrate genes). TF putatively binding *1-SST* are in red

Gene regulation underlying carbohydrate content variations were investigated through a weighted gene co-expression network analysis (WGCNA) using carbohydrate metabolic process (CMP, GO:0005975) and transcription factor (TF) genes (Table S7). Overall, six modules of co-expressed genes were identified (Fig. 3a) and organized into two large meta-modules (Fig. 3b) based on correlation relationships and named Meta1 (yellow, blue and brown modules) and Meta2 (turquoise, black and green). The modules showed a positive correlation within each meta-module and a negative correlation with those of the other meta-module. Moreover, Meta1 and Meta2 showed respectively negative and positive correlations with sugar amounts (Fig. 3c). Focusing on relationships between modules and carbohydrate contents, the highest positive correlation occurred between the turquoise one and kestose (*r* = 0.78, *p* = 1 × 10^−5^). The turquoise module consisted of 514 transcripts (Table S8) and included *1-SST* (CICEN011043.1), *1-FFT* (CICEN014948.1) and *1-FEHI* (CICEN009400.1). The significant positive correlation between *1-SST* and *1-FFT* and KES was previously observed in chicory^51^. The blue module showed the maximal negative correlation with KES (*r* = −0.77, *p* = 5 × 10^−5^) and with the turquoise module (*r* = −0.89, *p* = 2 × 10^−10^). The blue module consisted of 406 genes, including the chicory orthologues of CiMYB5 (CICEN021640.1) and CiMYB3 (CICEN012065.1), which are R2R3-MYB factors known to control *1-FEH*, *1-SST* and *1-FFT* in stress response^52^. Those transcripts with the highest correlation with KES (Fig. 3d), which were parametrized by gene significance (GS), showed the maximal centrality within the module as measured by module membership (MM). To downsize the number of candidate genes in KES regulation, transcripts of blue and turquoise modules were filtered by the simultaneous occurrence of highest GS, MM and intra-modular connectivity. The filtered transcripts formed the module genes of interest, named MGI. The turquoise-MGI consisted of 21 TFs and 10 genes of the carbohydrate metabolic process (Table S8) and this latter included *1-SST*. The positive correlation between turquoise-MGI *vs* KES contents and a prevailing trend of gene downregulation in waterlogged endives suggest that the module has an inductive role in KES accumulation. Conversely, the blue-MGI, which harboured 13 TFs and negative correlation *vs* KES amounts, showed a main gene upregulation that suggests a repressive function. Finally, the complexity of TF and carbohydrate genes relationships were depicted for blue- and turquoise-MGI in Fig. 3e.

#### Computational prediction of TFs controlling 1-SST

The *1-SST* repression strongly associated with KES content drop in waterlogged endives (Table S8), hence a trans-specific computational approach was used to identify candidate TFs (Table 3) acting on CICEN011043.1/*1-SST*. Given that the endive transcriptome just contains transcribed DNA, the promoter regions of *1-SST* from chicory and lettuce were used to score conserved binding sites (BS) known to be targeted by various TF classes, and those included in the blue and turquoise MGIs were reported. After filtering for BS n. ≥10 in the promoter of chicory *1-SST*, the best candidates were GATA (CICEN007985.1/GATA9 and CICEN020509.1/GATA15) and DOF (CICEN019145.1/CDF3 and CICEN025406.2/DOF3.4) families within the turquoise module, and WRKY types (CICEN030208.1/WRKY6 and CICEN011950.1/ WRKY75) in the blue group.

**Table 3:** Endive candidate TFs targeting 1-SST by scoring binding sites in promoters of chicory and lettuce orthologs

M	Endive transcription factors			Arabidopsis best hits		Binding sites n.	
	Transcript	Annotation	Type	AGI codes	Description	Chicory	Lettuce
T	CICEN007985.1	GATA9	GATA	AT4G32890.1	GATA transcription factor 9	36	22
T	CICEN005406.1	PIF1	bHLH	AT2G20180.7	Phytochrome interacting factor 1	4	2
T	CICEN010173.1	RAP2.7	AP2	AT2G28550.3	Related to AP2.7	1	1
T	CICEN016071.1	GATA11	GATA	AT1G08010.3	GATA transcription factor 11	3	4
T	CICEN019145.1	CDF3	DOF	AT3G47500.1	Cycling DOF factor 3	19	17
T	CICEN025406.2	DOF3.4	DOF	AT3G50410.1	DOF protein 3.4	19	15
T	CICEN013177.1	DEAR3	ERF	AT2G23340.1	DREB and EAR motif protein 3	2	4
T	CICEN020509.1	GATA15	GATA	AT3G06740.1	GATA transcription factor 15	35	20
B	CICEN031646.1	STZ	C2H2	AT1G27730.1	Salt tolerance zinc finger	3	3
B	CICEN030208.1	WRKY6	WRKY	AT1G62300.1	WRKY DNA-binding protein 6	23	8
B	CICEN011950.1	WRKY75	WRKY	AT5G13080.1	WRKY DNA-binding protein 75	10	4

NCBI acc. n. are in “Materials and methods”

*M* module, *T* turquoise, *B* blue

## Conclusions

Rainfall excess induced waterlog and affected the yield of four endive cultivars, which showed different stress-sensitivity, with the smooth-leafed ‘Flester’ having the lowest weight-loss. The NMR untargeted profiling enriched nutritive dataset with previously unreported compounds, was effective to highlight metabolic variations due to G, Y and GxY interaction and to address the inulin pathway. The enhancement of endive transcriptome allowed the identification of cultivar-specific (minimal in the least affected ‘Flester’) and cultivar-independent DEGs. These latter were enriched in GO terms consistent with those of leaves of waterlogged crops, and inclusive of carbohydrate metabolic process. The narrow down on the sucrose-inulin branch showed that the lowered expression of a sucrose gene set in parallel with that of *1-SST* accounted for sucrose and kestose contents drop of stressed leaves. WCGNA identified two anti-correlated modules harbouring candidate hub-genes, including *1-SST*, that may control positively and negatively kestose levels. BS computational analysis further supported that GATA, DOF and WRKY TFs might control *1-SST*.

## Materials and methods

### Plant material, growth conditions, and sampling

Curly-leafed ‘Domari’ and ‘Myrna’ and smooth ‘Confiance’ and ‘Flester’ cultivars (Enza Zaden Italy s.r.l.) respectively belong to *Cichorium endivia* var. *crispum* and *latifolium*; cultivations took place on the same parcel at comparable periods (September to November) in 2011 and 2012 with standardized interventions (Table S1). Soil characteristics are in Table S1, while soil humidity variation was monitored by tensiometers (mod. 8060, −60 to 0 kPa, probe length 30 cm by Stelzner/Pronova, Germany). Briefly, tensiometers were inserted (in pre-drilled holes) between 15 and 25 cm of depth and three measurements per week were carried out in three different points of the parcel hosting each cultivar. Figure 1 combines data on soil humidity, rainfall, relative humidity and temperature; the meteorological dataset was available on the public service web (http://dati.lazio.it/catalog/dataset/serie-storica-agrometeo). As for sampling, nine endive heads of each cultivar were selected according to market standards and weighted. Ten leaves (assumed as the target of consumption) were cut from each rosette and mixed to form a cultivar-specific pool that was subdivided into three distinct replicate batches (RB) of thirty leaves each. The RBs (of comparable weights) were frozen by liquid nitrogen, softly hand-crunched, and stored at −80 °C. Aliquots from each RB were directly used for RNA isolation or lyophilized at −50 °C for 72 h (lab freeze dryer, FreeZone®, Labconco Corp., Kansas City, MO, USA) and stored at −20 °C for NMR quantification.

### Metabolite extraction, NMR assignment and profiling

Lyophilized leaves were finely ground in liquid nitrogen using pre-cooled ceramic pestle and mortar. An amount of 25 mg was added to 0.90 mL of acetonitrile/water (1:1 v/v), the mixture was stirred for 30 s, centrifuged for 5 min (14,500 × *g*) and supernatant (0.74 mL) filtered through cotton wool in a glass vial. After solvent evaporation (N2 flux at room temperature), the residue was dissolved in 0.75 mL of 400 mM phosphate buffer (pH = 7) in D2O containing 1 mM 3-(trimethylsilyl)-propionic-2,2,3,3-d4 acid sodium salt (TSP) as an internal standard. The NMR spectra of aqueous extracts were recorded at 27 °C on a Bruker AVANCE 600 NMR spectrometer operating at the proton frequency of 600.13 MHz. TSP signal of methyl group (*d* = 0.00 ppm) was used as an internal standard for 1H spectra. Each 1H spectrum was acquired by co-adding 256 transients with a recycle delay of 3 s. The residual HDO signal was suppressed using a pre-saturation. The experiment was carried out by using a 45° pulse of 7.0 µs, 32 K data points. All the spectra were processed by means of the Bruker TOPSPIN software (version 1.3). After Fourier transformation, manual phase correction and baseline correction selected resonances in 1H NMR spectra (Table S3) were integrated to calculate metabolite concentrations. The integral value of TSP methyl groups (9H) was used as a reference for quantification. The content of selected metabolites was expressed as in mg g^-1^ on dry weight basis. 2D NMR experiments, namely 1H–1H total correlation spectroscopy (TOCSY), 1H–13C heteronuclear single quantum coherence (HSQC), and 1H–13C heteronuclear multiple bond correlation (HMBC), were performed using the same experimental conditions previously reported^53^. The mixing time for the 1H–1H TOCSY was 80 ms. The 1H–13C HSQC experiment was performed using a coupling constant 1JC–H of 150 Hz, whereas the 80 ms delay for the evolution of long-range couplings was used in 1H–13C HMBC experiments.

Carbohydrate contents were converted into SSE by using conversion values from literature (Table S4).

### Transcriptome assembly, annotation and identification of DEGs

The *Lactuca sativa* draft assembly (Lsat_Salinas_v7, GCA_002870075.1) was used as a reference genome to guide the endive transcriptome assembly. RNA from apices, stems, leaves, and roots of Domari cultivar at transplant and commercial maturation stages were sequenced as previously described^19^. The paired-end reads (NCBI-SRA: SRX3385280) were aligned to the genome by STAR v.2.7.0e (https://github.com/alexdobin/STAR/releases), partitioned according to locus, and assembled at each locus by Trinity v.2.8.2 (https://github.com/trinityrnaseq/trinityrnaseq/). Subsequently, the genome-guided and de novo^19^ transcriptomes (made of 80473 and 84882 transcripts, respectively) were combined to form a redundant set. The tr2aacds pipeline (http://arthropods.eugenes.org/EvidentialGene/trassembly.html) was used to generate a final non-redundant assembly (v.2). Potential coding sequences were first predicted using the TransDecoder software and then functionally annotated using Trinotate (https://github.com/Trinotate/Trinotate.github.io). The PlantTFDB v.4 (http://planttfdb.gao-lab.org/) was exploited to predict TFs. The quality assessment of transcriptome v.2 was carried out by (a) counting the reads that could be mapped back to the assembly as proper paired matches; (b) using BUSCO metrics (https://busco.ezlab.org/) to evaluate the assembly completeness based on the representation of near-universal single-copy orthologues; (c) assessing the number of nearly full (>70%) or full-length transcripts by the ‘blast_outfmt6_group_segments.pl’ script from the Trinity package; (d) aligning the transcripts against the latest lettuce genome release (GCA_002870075.2) by exploiting the ‘process_GMAP_alignments_gff3_chimeras_ok.pl’ script in Trinity package. The single-ends reads of all cultivars from Y1 and Y2 (NCBI Bioproject PRJNA417356) were aligned to the assembly v.2 and gene expression was quantified using Bowtie2 v 2.3.4.3 (http://bowtie-bio.sourceforge.net/bowtie2/index.shtml). For each cultivar, differential gene expression analysis between Y2 vs Y1 was carried out with the Bioconductor edgeR package and transcripts with false discovery rate (FDR) ≤ 0.05 and an absolute log_2_ fold change ≥1 were defined as DEGs. Quantitative PCR (qPCR) and normalization procedures were previously detailed^19^ by using the primers listed in Table S9.

### Network analysis

A co-expression gene network of TF and carbohydrate metabolic process (GO:0005975) genes was constructed using the WGCNA software package v1.68 (https://horvath.genetics.ucla.edu/html/CoexpressionNetwork/Rpackages/WGCNA/). We filtered out transcripts that were lowly expressed (RPKM < 1) in more than 70% of the libraries and had incomplete sequences (full-length ratios <80%) as compared to the respective NCBI blast hits; 2129 genes that satisfied these thresholds were retained. Normalized and log-transformed gene expression data were corrected for batch effect and the adjusted values were used for signed network construction and module detection. Briefly, soft thresholding power ß of 22 was chosen based on a scale-free topology approximation criterion, adjacency and dissimilarity based on topological overlap were computed. As for the detection of co-expressed gene clusters (modules), the Dynamic Tree cut algorithm (minimal module size of 30) and a branch merge cut height of 0.25 were used. The first principal component of each module (module eigengene, ME) was used to condense the gene expression variability within each module. MEs were exploited to study higher-order relationships among the modules and to relate gene co-expression patterns to carbohydrate amounts. The correlations between individual gene expressions and carbohydrate contents were reported as gene significance (GS) values. The correlation between a gene expression profile vs the ME (module membership, MM) was used to quantify how close a gene is to a given module. The sum of the adjacencies within a given module (intramodular connectivity, Kin) was calculated to measure the co-expression between a gene and other module members. As for the most significant modules vs kestose contents (‘Blue’ and ‘Turquoise’ modules), genes with the highest (top 10%) GS, MM and Kin were selected and those with a connection strength (edge weight) ≥0.2 were visualized with Cytoscape.

### Phylogenetic analyses and computational search of binding sites in gene promoters

Phylogenetic analysis was performed based on *Cichorieae* (*C. endive, C. intybus and L. sativa*) deduced proteins belonging to the glycoside hydrolase family 32 (GHF32). Fully- and nearly full length amino acid sequences from endive were selected those bearing the glyco_hydro_32N (PF00251) and glyco_hydro_32C (PF08244) domain annotations. Complete protein sequences from *C. intybus* and *L. sativa* were retrieved from NCBI protein db (access numbers are in Fig. S3 legend). ClustalW was used in multiple sequence alignments and the MEGA software (v. 7) running the neighbour-joining method allowed phylogenetic analyses and tree assembly. As for transcription binding site analysis, the 1070 base pair long genomic sequence upstream the ATG of the chicory (GenBank: EU545648.1) and lettuce (GenBank: CM022519.1, base location: 204758200..204759269) 1-SST orthologues were analyzed by PLANTPAN 3.0 (http://plantpan.itps.ncku.edu.tw/).

### Statistical analyses

All data (three biological and three analytical replicates) were analyzed according to a completely randomized design in a two-way ANOVA (genotypes × year of cultivation) by R studio script. The separation of means was obtained by Least Significant Difference (LSD) test. For visual analysis of the data, principal component analysis (PCA) was performed on mean centred and standardized data (unit variance scaled). The data matrix submitted to PCA was made of 8 observations (2 cultivation years × 4 genotypes) and 30 variables. The results were shown as biplots of scores (treatments) and loadings (variables) using R studio script. Pearson correlations were calculated using the “rcorr” function in the Hmisc package within the R environment (v 3.4.3).

## Supplementary information

Table S1

Table S2

Table S3

Table S4

Table S5

Table S9

Supplementary figures

Table S6

Table S7

Table S8
