# Supplementary material for: Leaf nutrient content and transcriptomic analyses of endive (Cichorium endivia) stressed by downpour-induced waterlog reveal a gene network regulating kestose and inulin contents
Source: Hortic Res. 2021 May 1;8:92. doi: 10.1038/s41438-021-00513-2 (PMC8087766; doi:10.1038/s41438-021-00513-2)
Supplement: Supplementary file 1 — Table S1 [file 41438_2021_513_MOESM1_ESM.docx]

## **Table S1. Site, soil characteristics and cultivation**

| **Cultivation site** |  | | |
| --- | --- | --- | --- |
| Location | Tarquinia, Viterbo, Lazio (Italy) | | |
| Coordinates | 42°16N 11°42'E | | |
| Altitude | 31 m a.s.l | | |
| **Soil parameters** | | | |
| USDA soil classification | clay loam |  | |
| Sand (2-0.05 mm) | 44% |  | |
| Silt (0.05-0.002 mm) | 21% |  | |
| Clay (<0.002 mm) | 35% |  | |
| pH | 7.0 |  | |
| Electr. cond. | 0.163 mS cm^-1^ |  | |
| CaC03 | traces |  | |
| Organic matter | 11.3 g kg^-1^ |  | |
| Total nitrogen | 0.75 g kg^-1^ |  | |
| P2O5 available | 23 mg kg-1 |  | |
| Fe available | 20.6 mg kg^-1^ |  | |
| Mn available | 12.2 mg kg^-1^ |  | |
| Cu available | 2.0 mg kg^-1^ |  | |
| Zn available | 0.8 mg kg^-1^ |  | |
| Ca exchange | 2250 mg kg^-1^ |  | |
| Mg exchange | 474 mg kg^-1^ |  | |
| K exchange | 411 mg kg^-1^ |  | |
| Na exchange | 85 mg kg^-1^ |  | |
| C.E.C. | 16.6 meq 100g-1 |  | |
| **Field operation** | **Notes** | **2011** | **2012** |
| Basal dressing. | Nitrophoska special 12.12.7 (EuroChem Agro), 500 kg ha^-1^ | 12/08 | 10/08 |
| Protection vs thrips. | Success (Bayer), a.p. spinosad, 0.25 L ha^-1^ | 20/09 | 24/09 |
| Fertirrigation. | Ammonium sulphate, 100 kg ha^-1^ | 29/09 | 02/10 |
| Protection vs fungi | Signum (Basf), pa. boscalid + pyraclostrobin,1 kg ha^-1^ | 07/10 | 10/10 |
| Protection vs thrips | see above | 07/10 | 10/10 |
| **Cultivation stages** | **Notes** | **2011** | **2012** |
| Sowing | nursery, substrate, 3 dm^2^/well | 23/08 | 23/08 |
| Transplant | open field, 8.2 plants/m^2^, 0.35x0.35m | 14/09 | 17/09 |
| Harvest | transport and storage at 7°C, dark | 17/11 | 16/11 |
| Sampling | storage at -80°C or at -20°C post freeze-drying | 18/11 | 16/11 |
