# Supplementary material for: Leaf nutrient content and transcriptomic analyses of endive (Cichorium endivia) stressed by downpour-induced waterlog reveal a gene network regulating kestose and inulin contents
Source: Hortic Res. 2021 May 1;8:92. doi: 10.1038/s41438-021-00513-2 (PMC8087766; doi:10.1038/s41438-021-00513-2)
Supplement: Supplementary file 2 — Table S2 [file 41438_2021_513_MOESM2_ESM.docx]

## **Tab. S2**. Rain data during 2011 and 2012

| **Parameters** | **2011** | **2012** | **Δ (%)** |
| --- | --- | --- | --- |
| Rain (mm) - Transp. to Harvest | 67.80 | 225.80 | +233 |
| Rain (mm) - 15 days before H | 33.20 | 114.60 | +245 |
| Mean Daily Rain (mm) - T to H | 1.04 | 3.70 | +255 |
| MDR - 15 days before H | 1.95 | 7.64 | +291 |
| Number of Dry Days | 56 | 43 | -23 |
| Number of Rainy Days | 9 | 18 | +100 |
| Rain frequency (%) | 13.84 | 29.5 | +113 |
