# Supplementary material for: Leaf nutrient content and transcriptomic analyses of endive (Cichorium endivia) stressed by downpour-induced waterlog reveal a gene network regulating kestose and inulin contents
Source: Hortic Res. 2021 May 1;8:92. doi: 10.1038/s41438-021-00513-2 (PMC8087766; doi:10.1038/s41438-021-00513-2)
Supplement: Supplementary file 3 — Table S3 [file 41438_2021_513_MOESM3_ESM.docx]

**Table S3.** Metabolites identified by NMR analysis of aqueous extracts of endive leaves.

| **Compound** | **Assignment** | | **^1^H**  **(ppm)** | | **Multiplicity [J(Hz)]** | **^13^C**  **(ppm)** |
| --- | --- | --- | --- | --- | --- | --- |
| *Carbohydrates* | | | | | | |
| α-Glucose(α-GLC) | | CH-1 | 5.25*^a^* | | d [3.8] | 93.1 |
|  | | CH-2 | 3.55 | |  | 72.2 |
|  | | CH-3 | 3.72 | |  | 73.7 |
|  | | CH-4 | 3.42 | | t [9.2] | 70.6 |
|  | | CH-5 | 3.84 | |  | 72.4 |
|  | | CH_2_- 6,6’ | 3.84,3.78 | |  | 61.5 |
| β-Glucose(β-GLC) | | CH-1 | 4.66*^a^* | | d [7.9] | 96.9 |
|  | | CH-2 | 3.26 | | dd[9.3;8.0] | 75.1 |
|  | | CH-3 | 3.50 | | t [9.1] | 76.7 |
|  | | CH-4 | 3.42 | | dd[9.7;9.2] | 70.6 |
|  | | CH-5 | 3.47 | |  | 76.9 |
|  | | CH_2_-6,6’ | 3.90, 3.74 | |  | 61.7 |
| β-D-Fructofuranose(β-FRUfu) | | CH_2_-1,1’ | 3.61, 3.57 | |  | 63.7 |
|  | | C-2 |  | |  | 102.5 |
|  | | CH-3 | 4.12^a^ | | m | 76.4 |
|  | | CH-4 | 4.12^a^ | | m | 75.4 |
|  | | CH-5 | 3.84 | |  | 81.6 |
|  | | CH_2_-6,6’ | 3.82,3.68 | |  | 63.3 |
| α-D-Fructofuranose (α-FRUfu) | | CH-3 | 4.13^a^ | |  | 82.9 |
|  | | CH-5 | 4.07 | |  | 82.3 |
| β-D-Fructopyranose(β-FRUpy) | | CH_2_-1,1’ | 3.72,3.57 | | d [2.7] | 64.8 |
|  | | CH-3 | 3.81 | |  | 68.5 |
|  | | CH-4 | 3.90 | |  | 70.6 |
|  | | CH-5 | 4.01 | |  | 70.1 |
|  | | CH_2_-6,6’ | 4.03, 3.71 | |  | 64.3 |
| Sucrose(SUCR) | | CH-1 (Glc) | 5.42*^a^* | | d [3.8] | 93.2 |
|  | | CH-2 | 3.57 | |  | 72.1 |
|  | | CH-3 | 3.77 | |  | 73.6 |
|  | | CH-4 | 3.48 | |  | 70.2 |
|  | | CH-5 | 3.85 | |  | 73.3 |
|  | | CH_2_-6 | 3.83 | |  | 61.1 |
|  | | CH_2_-1’(Fru) | 3.69 | |  | 62.3 |
|  | | C-2’ |  | |  | 104.7 |
|  | | CH-3’ | 4.22 | | d [8.7] | 77.4 |
|  | | CH-4’ | 4.06 | |  | 75.0 |
|  | | CH-5’ | 3.90 | |  | 82.3 |
|  | | CH_2_-6’ | 3.83 | |  | 63.3 |
| Kestose/Inulin | | CH-1(Glc) | 5.44*^ab^* | | d [3.7] | 93.5 |
|  | | CH-2 | 3.56 | |  | 72.1 |
|  | | CH-3 | 3.77 | |  | 73.5 |
|  | | CH-4 | 3.48 | |  | 70.2 |
|  | | CH-5 | 3.85 | |  | 73.4 |
|  | | CH_2_-6 | 3.83 | |  | 61.1 |
|  | | C-2’ (Fru) |  | |  | 104.6 |
|  | | CH-3’a | 4.20 | |  | 77.6 |
|  | | CH-3’b | 4.28 | |  | 77.7 |
| *Organic acids* | | | | | | |
| Citric Acid (CA) | | α,γ-CH | 2.54*^a^* | | d[15.3] | 46.6 |
|  | | α’,γ’-CH | 2.67 | | d[15.3] | 46.6 |
| Fumaric Acid (FA) | | α-CH=CH | 6.52*^a^* | | s |  |
| Lactic Acid (LA) | | β- CH_3_ | 1.33*^a^* | | d[6.9] | 21.1 |
|  | | α- CH | 4.12 | |  |  |
| Malic Acid (MA) | | α- CH | 4.30*^a^* | | dd[9.9;3.2] | 71.3 |
|  | | β- CH | 2.68 | | dd[15.4;3.2] | 43.6 |
|  | | β’- CH | 2.38 | | dd[15.4;9.9] | 43.6 |
| Succinic Acid (SA) | | α,β-CH_2_ | 2.41*^a^* | | s | 35.1 |
| Tartaric Acid (TA) | | CH | 4.34*^a^* | | s | 74.9 |
| *Polyols* | | | | | | |
| *Chiro*-inositol(CI) | | CH-1,6 | | 4.04 |  | 72.6 |
|  | | CH-2,5 | | 3.76 |  | 71.4 |
|  | | CH-3,4 | | 3.59*^a^* |  | 73.7 |
| *Myo*-inositol(MI) | | CH-1 | | 4.08 |  | 73.2 |
|  | | CH-2,5 | | 3.54 |  | 72.4 |
|  | | CH-3,6 | | 3.63 |  | 73.4 |
|  | | CH-4 | | 3.29*^a^* | t [9.3] | 75.3 |
| *Scyllo*-inositol(SI) | | CH-1,6 | | 3.36*^a^* | s | 74.6 |
| Quinic Acid (QA) | | CH_2_-1,1’ | 1.88*^a^* 2.07 | | dd[13.3;10.9] | 41.7  41.7 |
|  | | CH-2 | 4.02 | |  | 67.9 |
|  | | CH-3 | 3.56 | |  | 76.3 |
|  | | CH-4 | 4.15 | | q [3.5] | 71.4 |
|  | | CH_2_-5,5’ | 2.04, 1.97 | |  | 38.3 |
| *Amino acids* | | | | | | |
| Alanine (ALA) | | α-CH | | 3.78 |  |  |
|  | | β-CH_3_ | | 1.49*^a^* | d [7.3] |  |
| Asparagine (ASN) | | α-CH | | 4.01 |  | 52.2 |
|  | | β-CH | | 2.89 |  | 35.6 |
|  | | β’-CH | | 2.96*^a^* | dd [16.9;7.3] | 35.6 |
| Aspartic Acid (ASP) | | α-CH | | 3.90 |  | 53.1 |
|  | | β-CH | | 2.81*^a^* | dd [17.5;3.8] | 37.5 |
|  | | β’-CH | | 2.71 | dd [17.5;8.4] | 37.5 |
| γ-Aminobutyric Acid (GABA) | | α-CH_2_ | | 2.30*^a^* | t [7.5] | 35.4 |
|  | | β-CH_2_ | | 1.91 |  | 24.6 |
|  | | γ-CH_2_ | | 3.02 |  | 40.2 |
| Glutamic Acid (GlU) | | α-CH | | 3.76 |  | 55.6 |
|  | | β-CH_2_ | | 2.12, 2.07 |  | 27.9 |
|  | | γ-CH_2_ | | 2.35*^a^* |  | 34.5 |
| Glutamine (GLN) | | α-CH | | 3.78 | t [6.2] | 55.1 |
|  | | β-CH_2_ | | 2.15 |  | 27.2 |
|  | | γ-CH_2_ | | 2.46*^a^* |  | 31.8 |
| Isoleucine (ILE) | | α-CH | | 3.68 |  | 60.5 |
|  | | β-CH | | 1.99 |  | 36.9 |
|  | | γ-CH_2_ | | 1.48, 1.27 |  | 25.5 |
|  | | γ-CH_3_ | | 1.01*^a^* | d [7.1] | 15.6 |
|  | | δ-CH_3_ | | 0.95 | t [7.4] | 12.1 |
| Phenylalanine (PHE) | | CH-2,6 | | 7.34 |  | 130.4 |
|  | | CH-3,5 | | 7.44*^a^* | t[7.3] | 130.1 |
|  | | CH-4 | | 7.39 |  |  |
| Threonine (THR) | | α-CH | | 3.60 |  |  |
|  | | β-CH | | 4.12 |  |  |
|  | | γ-CH_3_ | | 1.33*^a^* | d [6.4] | 20.4 |
| Valine (Val) | | α-CH | | 3.62 |  | 61.3 |
|  | | β-CH | | 2.28 |  | 30.0 |
|  | | γ-CH_3_ | | 0.99 | d [7.1] | 17.7 |
|  | | γ’-CH_3_ | | 1.04*^a^* | d [7.1] | 18.9 |
| *Phenols* | | | | | | |
| Chicoric Acid (CHA) | | CH(O)COOH | | 5.54*^a^* | s | 75.6 |
|  | | =CH-COO− | | 6.48 | d [15.8] | 115.1 |
|  | | −CH= | | 7.73 | d [15.8] | 147.7 |
|  | | CH-2’ | | 7.26 | d [2.1] | 116.3 |
|  | | CH-5’ | | 6.97 | d [8.2] | 117.3 |
|  | | CH-6’ | | 7.17 | dd [8.2;2.1] | 123.8 |
| Mono-caffeoyl-tartaric acid | | CH(OH)COOH | | 4.55*^a^* | d [2.2] | 73.8 |
| (MCTA) | | CH(O)COOH | | 5.30 | d [2.2] | 77.7 |
|  | | =CH-COO− | | 6.43 | d [16.1] |  |
|  | | −CH= | | 7.67 | d [16.1] |  |
|  | | CH-2’ | | 7.24 | d [2.1] |  |
|  | | CH-5’ | | 6.97 | d [8.1] |  |
| *Miscellaneous* | | | | | | |
| Choline (CHN) | | N(CH_3_)_3_^+^ | 3.21*^a^* | | s | 54.9 |
|  | | α-CH_2_ | 3.53 | |  | 68.4 |
| Ethanolamine (ETA) | | CH_2_-1 | 3.15*^a^* | | t [5.2] | 42.2 |
|  | | CH_2_-2 | 3.83 | |  | 58.6 |

^a^ Signals used for integration.

^b^ The signals of CH-1(Glc) group from kestose and inulin were partially overlapped. To separate the integrals a standard deconvolution procedure of Bruker TOPSPIN 1.3 software was applied.
