# Supplementary material for: Leaf nutrient content and transcriptomic analyses of endive (Cichorium endivia) stressed by downpour-induced waterlog reveal a gene network regulating kestose and inulin contents
Source: Hortic Res. 2021 May 1;8:92. doi: 10.1038/s41438-021-00513-2 (PMC8087766; doi:10.1038/s41438-021-00513-2)
Supplement: Supplementary file 4 — Table S4 [file 41438_2021_513_MOESM4_ESM.docx]

## **Table S4.** Sucrose Sweetness Equivalency

|  | **Sucrose Sweetness Equivalency (SSE)** | | | | | | | | **Variation**  **sources** | | |
| --- | --- | --- | --- | --- | --- | --- | --- | --- | --- | --- | --- |
|  | **2011** | | | | **2012** | | | |  |  |  |
| **Comp.**^a^ | **Domari** | **Myrna** | **Confiance** | **Flester** | **Domari** | **Myrna** | **Confiance** | **Flester** | **G** | **Y** | **GxY** |
| αGLC | 29.9±7.3 | 30.2±3.9 | 30.1±4.7 | 28.1±5.1 | 23.6±3.1 | 14.0±3.1 | 19.2±5.7 | 12.0±3.2 | *ns* | ***** | *** |
| βGLC | 57.0±13.0 | 56.8±7.2 | 56.6±8.5 | 53.4±10.1 | 45.8±6.9 | 26.6±6.1 | 36.8±11.0 | 23.1±5.5 | *ns* | ***** | **** |
| FRU | 259.4±62.4 | 253.1±32.9 | 254.8±34.1 | 249.6±45.7 | 235±29.4 | 164.5±37.8 | 196.4±55.9 | 147.8±30.7 | *ns* | **** | *ns* |
| INUL | 0.3±0.0 | 0.2±0.0 | 0.2±0.0 | 0.1±0.0 | 0.2±0.1 | 0.1±0.0 | 0.1±0.0 | 0.1±0.0 | ***** | ***** | *** |
| KES | 3.0±0.8 | 3.0±0.7 | 1.5±0.4 | 1.5±0.3 | 1.1±0.5 | 0.5±0.3 | 0.3±0.2 | 0.7±0.3 | ***** | ***** | ***** |
| SUC | 34.7±6.0 | 25.2±4.3 | 28.7±6.6 | 22.5±2.2 | 16.2±3.5 | 16.1±4.2 | 9.6±3.1 | 17.9±3.8 | ***** | ***** | ***** |
| Total | 384.2±84.2 | 368.5±46.5 | 372.0±50.0 | 355.2±62.0 | 322.0±40.3 | 221.7±47.0 | 262.3±75.5 | 201.5±38.9 | *ns* | ***** | *ns* |

a, Compounds. Glucose (GLC), **0.74**; fructose (FRU), **1.75**; sucrose (SUC), **1.00**; KES (GFn+Fn, 2≤n≤7), **0.35**; inulin (INUL; GFn, 2≤n≤60) **0.1** (ref. ^1,2^)

**References**

1 Franck, A. Technological functionality of inulin and oligofructose. *British Journal of Nutrition* **87**, S287-S291 (2007).

2 Shallenberger, R. S. in *Taste Chemistry* (ed Robert S. Shallenberger) 189-212 (Springer US, 1993).
