# Supplementary material for: Leaf nutrient content and transcriptomic analyses of endive (Cichorium endivia) stressed by downpour-induced waterlog reveal a gene network regulating kestose and inulin contents
Source: Hortic Res. 2021 May 1;8:92. doi: 10.1038/s41438-021-00513-2 (PMC8087766; doi:10.1038/s41438-021-00513-2)
Supplement: Supplementary file 5 — Table S5 [file 41438_2021_513_MOESM5_ESM.docx]

**Table S5. Nitrate contents (mg/kg FW) in leaves**

| **Genotype** | **2011** | **2012** | **Loss (%)** |
| --- | --- | --- | --- |
| Domari | 1433.4±249.8 | 941.0±146.3 | -34.35 |
| Myrna | 2194.7±404.7 | 1332.0±193.5 | -39.31 |
| Confiance | 1685.0±307.3 | 1759.7±148.2 | 4.43 |
| Flester | 2335.4±379.7 | 1357.3±267.8 | -41.88 |
