# Supplementary material for: Leaf nutrient content and transcriptomic analyses of endive (Cichorium endivia) stressed by downpour-induced waterlog reveal a gene network regulating kestose and inulin contents
Source: Hortic Res. 2021 May 1;8:92. doi: 10.1038/s41438-021-00513-2 (PMC8087766; doi:10.1038/s41438-021-00513-2)
Supplement: Supplementary file 6 — Table S9 [file 41438_2021_513_MOESM6_ESM.docx]

**Table S9.** Primer list

| **ID** | **Annotation** | **Symbol** | **Forwad/Reverse primers (5’-3’)** |
| --- | --- | --- | --- |
| **CICEN011043.1** | sucrose:sucrose 1-fructosyl transferase | *1-SST* | TCTGGTTGCTGTTATCCACGAC  CACAAGTTGGTCTCAAACGGGT |
| **CICEN014948.1** | fructan-fructan 1-fructosyltransferase | *1-FFT* | ATCCAACCGCACTCCCCTACTA  GTTCAGGACGATGAGCACGAAA |
| **CICEN009400.1** | fructan 1-exohydrolase I | *1-FEHI* | TGGACAATTCCGAGATCCATCA  GTCAGGACACTCCCACATACCC |
| **CICEN017634.1** | fructan 1-exohydrolase IIb | *1-FEHIIb* | TTGGGACTTACAGTCCTGATCG  AAACCCTTCTGTTCTTGGCATC |
| **EF528575** | Actin | *ACT* | CCAAATCCAGCTCATCAGTCG  TCTTTCGGCTCCGATGGTGAT |
