# Supplementary material for: Leaf nutrient content and transcriptomic analyses of endive (Cichorium endivia) stressed by downpour-induced waterlog reveal a gene network regulating kestose and inulin contents
Source: Hortic Res. 2021 May 1;8:92. doi: 10.1038/s41438-021-00513-2 (PMC8087766; doi:10.1038/s41438-021-00513-2)
Supplement: Supplementary file 7 — Supplementary figures [file 41438_2021_513_MOESM7_ESM.pdf]

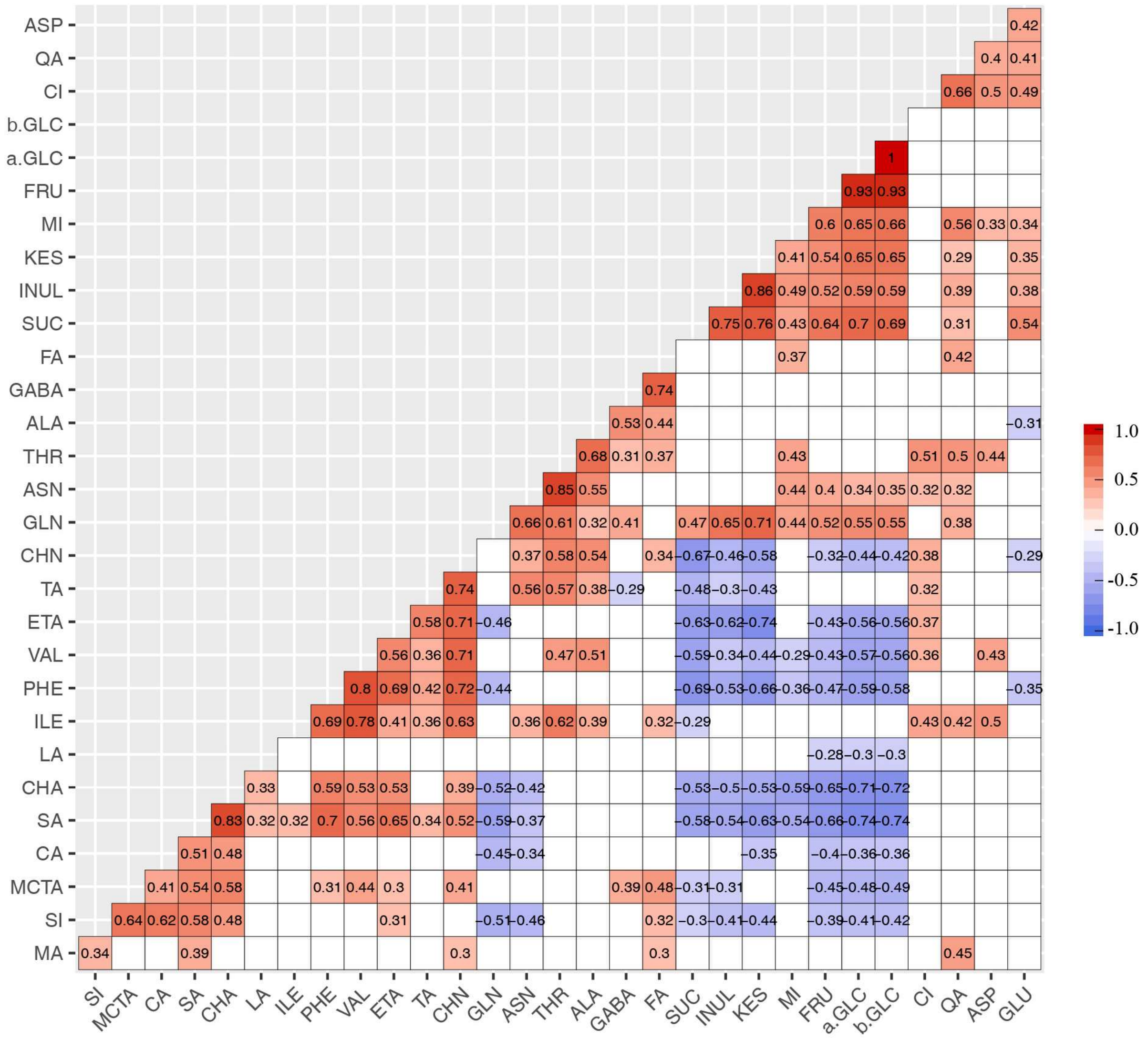

Figure S1. Correlation plot among metabolites. Significant Pearson's correlation coefficients (r≤ 0.01) are reported into the boxes, whilst the non-significant ones were omitted and boxes left blank. Heatmap is used to indicate the strength of correlation between the variables with positive and negative values are red and blue, respectively. FA, Fumaric acid; ASN, Asparagine; ASP, Aspartic acid; CA, Citric acid; ALA, Alanine; ETA, Ethanolamine; FRU, Fructose; GABA, γ-Aminobutyric acid; GLC, Glucose; GLN, Glutamine; GLU, Glutamic acid; ILE, Isoleucine; INUL, inulin; KES, Kestose; LA, Lactic acid; MA, Malic acid; MCTA, Monocaffeoyl tartaric acid; MI, Myo-inositol; CI, Chiro-inositol; PHE, Phenylalanine; CHN, Choline; CHA, Chicoric acid; QA, Quinic acid; SA, Succinic acid; SI, Scyllo-inositol; SUC, Sucrose; TA, Tartaric acid; THR, Threonine; VAL, Valine.

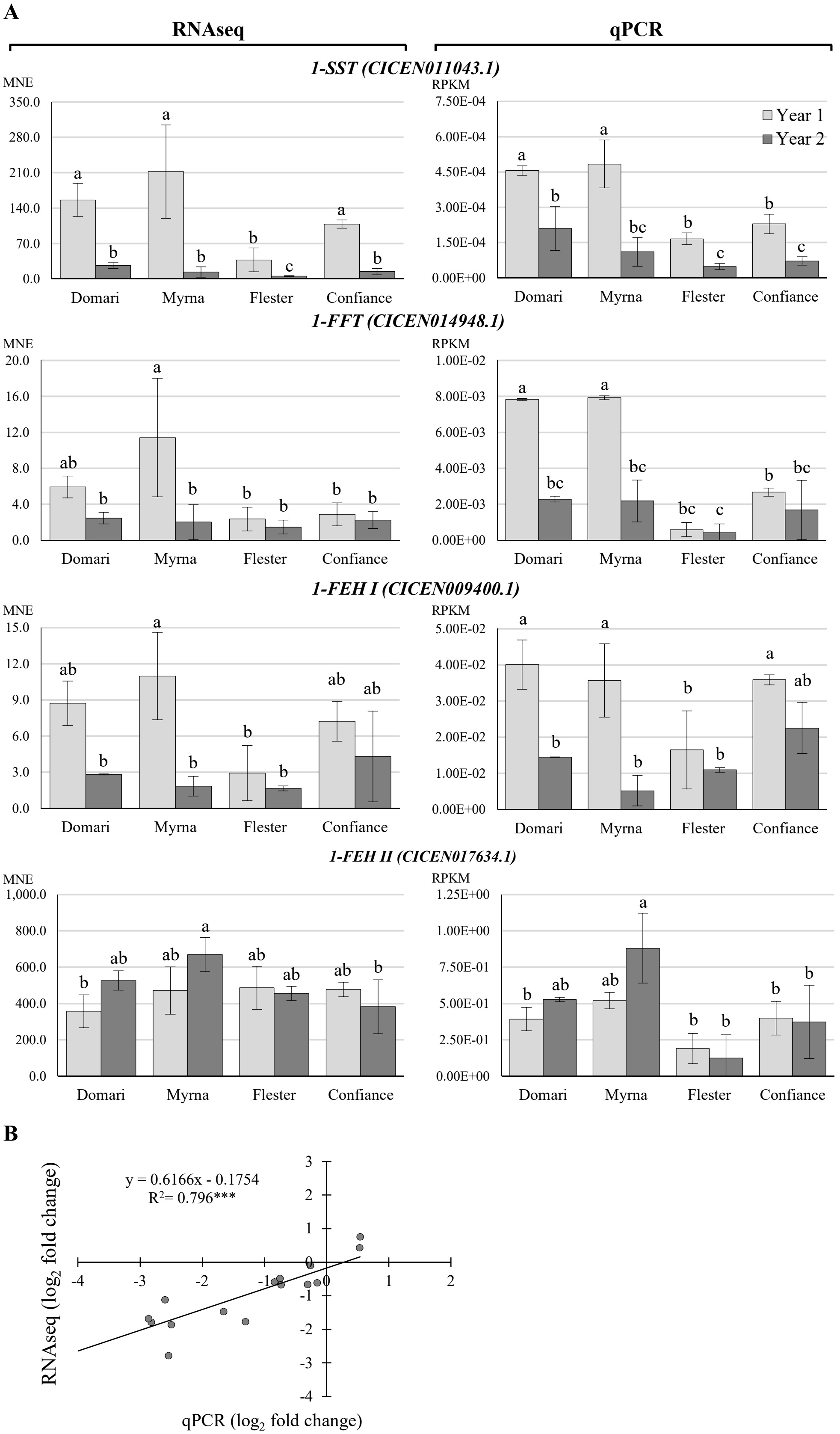

Figure S2. Validation of gene expression patterns. A) The RNAseq expression profiles (left column) of four genes belonging to the KES and INUL pathway were checked by quantitative-PCR (right column) in four *C. endivia* cultivars grown in Y1 and Y2 cycles. The expression levels of the target genes were normalized with the reference genes *ACT*. Bars marked with the same letters were not significantly different after the ANOVA and HSD Tukey's test. MNE, mean normalised expression; RPKM, reads per kilo base per million mapped reads. B) Correlation of differential expression ratios (log2 fold change) obtained from RNAseq and qPCR analyses. For a given unigene, RNAseq fold change refers to the ratios of RPKM values of Y2 vs Y1, whilst qPCR fold change is the relative expression of Y2 normalized to those of Y1. Significant positive correlation occurred between the expression fold changes measured by the two methods (R^2 = 0.796; $P < 0.001$).

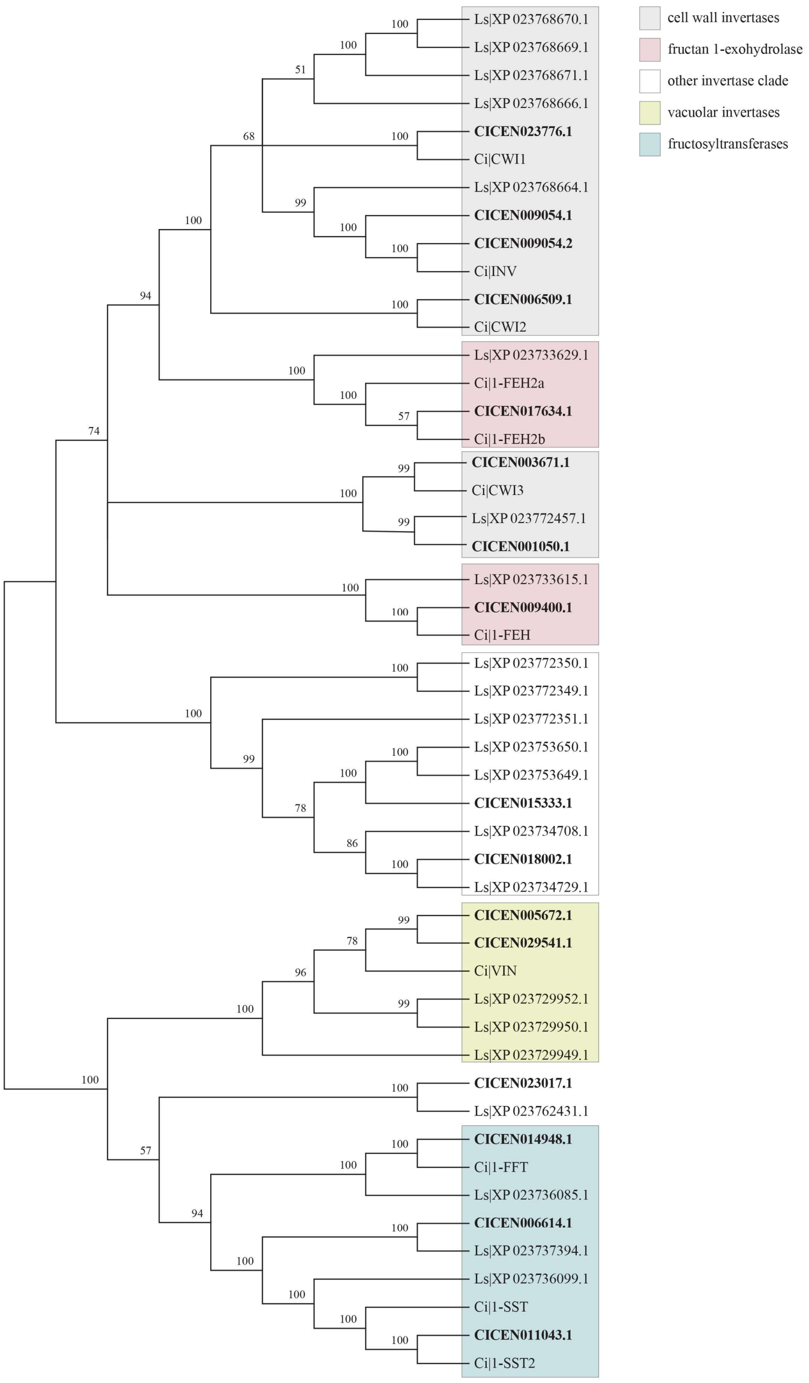

Figure *S3*. Phylogenetic trees of glycoside hydrolase family 32 (GHF32). Tree was inferred with the neighbor-joining method and the bootstrap values are based on 1000 replicates. The analysis involved amino acid sequences of the *Cichorieae* tribe and those from *Cichorium endivia* are in bold. Colored boxes indicate the different enzyme subgroups. As for *C. intybus* (Ci) sequences, NCBI refseq accession number are: AIP90174.1 (1-FEH2b|Ci), AAD00558.1 (1-FFT|Ci), ARO49602.1 (VIN|Ci), AAB58909.1 (1-SST|Ci), AFB83198.1 (1-SST2|Ci), AAP85536.1 (1-FEH2a|Ci), CAC19366.1 (1-FEH|Ci), CAA72009.1 (INV|Ci), AQR55698.1 (CWI1|Ci), AQR55697.1 (CWI2|Ci), ARO49601.1 (CWI3|Ci). As for *Lactuca sativa* (Ls), the NCBI refseq accession number are: XP_023737394.1, XP_023736099.1, XP_023736085.1, XP_023734729.1, XP_023734708.1, XP_023733629.1, XP_023733615.1, XP_023729952.1, XP_023729950.1, XP_023729949.1, XP_023772351.1, XP_023772350.1, XP_023772349.1, XP_023768671.1, XP_023768670.1, XP_023768669.1, XP_023768666.1, XP_023768664.1, XP_023762431.1, XP_023753650.1, XP_023753649.1, XP_023772457.1.

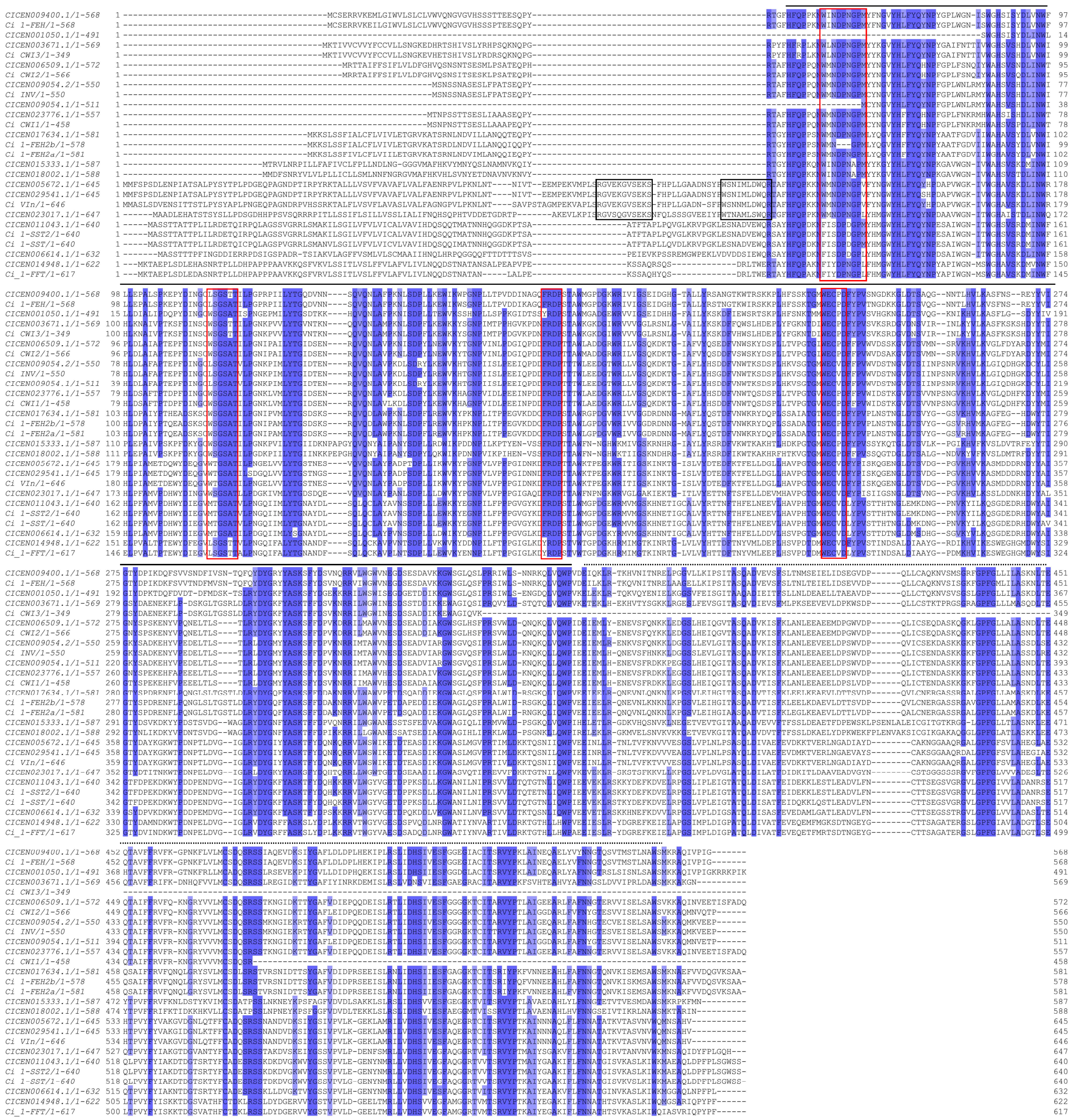

Figure S4. Comparison of conserved amino acid motifs specific to GHF32 proteins from *C. endive* and *C. intybus*. Residues sharing an identiy threshold of 70% were purple highlighted. The N- (PF00251) and C-terminal (PF08244) domains featuring the glycosyl hydrolase family 32 are respectively reported as solid and dotted lines above the alignment. Red boxes mark the conserved motifs (WMNDPNG, WSGSAT, RDP and EC domains) in the active sites. Black boxes indicate typical vacuolar invertase sequences (Van den Ende, 2002)
